# Novel Freshwater Ascomycetes from Submerged Plant Debris in the Zújar River (Extremadura Community, Spain)

**DOI:** 10.3390/jof12020102

**Published:** 2026-01-31

**Authors:** María Barnés-Guirado, Alberto Miguel Stchigel, José Francisco Cano-Lira

**Affiliations:** Mycology Unit, Medical School, Universitat Rovira i Virgili, C/Sant Llorenç 21, 43201 Reus, Spain; maria.barnes@estudiants.urv.cat (M.B.-G.); jose.cano@urv.cat (J.F.C.-L.)

**Keywords:** biodiversity, freshwater, fungi, phylogeny, taxonomy

## Abstract

Freshwater fungi remain insufficiently documented in the Mediterranean river systems despite their key roles in organic-matter turnover. Here, we surveyed filamentous fungi associated with submerged decaying plant debris in the Zújar River (Extremadura, southwestern Spain) using a culture-based approach combined with phenotypic characterization and multilocus phylogenetic analyses (ITS, LSU, *rpb*1, *rpb*2 and *tef-*1α). A total of 49 strains were isolated and identified, revealing a diverse assemblage of Ascomycota. Five taxa are described as new to science: *Arachnopeziza torrehermosensis*, *Conioscypha clavatispora*, *Neoanungitea torrehermosensis*, *Ophioceras diversisporum* and *Polyscytalum submersum*. Notably, *Polyscytalum submersum* represents the first record of the genus for the Iberian Peninsula, while *Arachnopeziza torrehermosensis*, *Neoanungitea torrehermosensis* and *Ophioceras diversisporum* constitute the first records of their respective genera for Spain (and *Neoanungitea torrehermosensis* also for Europe). In addition, phylogenetic evidence supports taxonomic refinements within the orders *Magnaporthales* and *Conioscyphales*, including the establishment of *Protophioceras* to accommodate *Ophioceras sichuanense* and the establishment of *Protoconioscypha* for two previously misclassified *Conioscypha* species. Overall, this first mycological report of submerged plant debris in the Zújar River substantially expands knowledge of freshwater fungal diversity in the region and provides a refined framework for the taxonomy of several lineages of aquatic-associated ascomycetes.

## 1. Introduction

Freshwater ecosystems cover only ~0.8% of the Earth’s surface and account for ~0.01% of global water volume, yet they host nearly 7% of global biodiversity [1,2]. Despite their ecological importance, these habitats are increasingly threatened by climate change, hydrological alterations, pollution, and other anthropogenic pressures. In the Iberian Peninsula, some rivers follow a Mediterranean hydrological regime characterized by seasonal variability, including flash floods and prolonged summer droughts [3], while reservoirs and artificial wetlands constitute most lentic systems due to extensive hydraulic infrastructure [4].

Fungi constitute an essential but understudied inhabitant of freshwater ecosystems, where they participate in nutrient cycling, organic matter decomposition, and symbiotic interactions [5]. Taxonomically, freshwater fungi span several phyla, with Ascomycota being the most diverse group [6]. They are usually classified into four ecological groups: Ingoldian, aero-aquatic, terrestrial-aquatic, and submerged-aquatic fungi [6]. Ingoldian fungi, which sporulate underwater and often produce distinctive scolecoid or stauroid conidia, are particularly abundant in lotic systems, where they play a central role in decomposing allochthonous plant material [7,8]. Aero-aquatic fungi, in contrast, sporulate only after exposure to air and typically inhabit lentic environments [5,9]. Other groups, such as terrestrial-aquatic and submerged-aquatic fungi, colonize plant material at the water–land interface or in submerged substrates [10,11,12].

The Zújar River, a 215 km tributary of the Guadiana Basin in southwestern Spain, drains ~8500 km^2^ under a Mediterranean climate marked by low rainfall (<500 mm/year) and pronounced seasonal fluctuations in discharge [13,14,15]. Its riparian vegetation includes *Populus alba*, *P. nigra*, *Fraxinus angustifolia* and *Salix* spp., together with shrubs such as *Nerium oleander* and *Tamarix africana*. Aquatic vegetation comprises submerged (*Ceratophyllum demersum*, *Potamogeton* spp.), floating (*Lemna minor*, *Ranunculus aquatilis*) and emergent taxa (*Carex* spp., *Phragmites australis*, *Typha latifolia*) [13,16,17,18,19]. The river crosses intensively cultivated landscapes dominated by cereals and olives, and its course has been heavily modified by reservoirs and irrigation channels [14,20].

Although the flora and aquatic fauna of the Zújar River and its basin have been studied, information on its microbial and, particularly, fungal diversity remains unexplored. The present study addresses this gap by investigating freshwater ascomycetes associated with submerged plant debris in the Zújar River using culture-dependent isolation and a polyphasic taxonomic approach, and by describing novel taxa that contribute to our understanding of the freshwater mycobiota of Mediterranean river systems.

## 2. Materials and Methods

### 2.1. Sampling and Fungal Isolation

Thirty-one samples of submerged decomposing plant material (leaves and wood) were collected in autumn 2022 from the Zújar River along a transect ranging from 38°25′13.9″ N, 5°34′36.5″ W to 38°25′58.5″ N, 5°34′15.6″ W (BA-159). Samples were placed in sterile self-sealing plastic bags, transported to the laboratory at room temperature (20–25 °C), and stored at 4 °C until processing. Samples were rinsed two to five times, depending on the amount of sediment present, by adding 500 mL of tap water to the transport bags. After rinsing, each sample was placed in 90 mm-diameter Petri dishes lined with three layers of sterile filter paper moistened with sterile distilled water and incubated in the dark at room temperature. Fungal development was monitored daily for up to 2 months using a stereomicroscope (Leica Microsystems, model EZ4, Wetzlar, Germany). Fertile structures (mostly) or hyphal tips were transferred using sterile disposable tuberculin-type needles to oatmeal agar medium (OA; 15 g filtered oat flakes and 7.5 g agar in 500 mL tap water [21]) in 50 mm-diameter Petri dishes. The OA plates were incubated under the same conditions, and transfers were repeated until axenic cultures of each isolate were obtained. Fungal strains representing scarcely reported taxa and putative novel species were deposited in the culture collection of the Faculty of Medicine of Reus (FMR; Reus, Tarragona Province, Spain). Ex-type strains and holotype specimens (dried cultures) were deposited at the Westerdijk Fungal Biodiversity Institute (CBS; Utrecht, the Netherlands).

### 2.2. Phenotypic Study

The macroscopic characterization of the colonies was performed on potato carrot agar (PCA; 10 g potato, 10 g carrot, 6.5 g agar, 500 mL distilled water [22]), potato dextrose agar (PDA; Laboratorios Conda S.A., Madrid, Spain), OA and 2% malt extract agar (MEA; Difco Inc., Detroit, MI, USA) after incubation in darkness at 25 °C for 14 days. The cardinal growth temperatures of each strain of interest were determined on PDA medium at 5–40 °C in 5 °C intervals, with an additional measurement at 37 °C.

Microscopic characterization was carried out by growing the fungal strains on OA at 25 °C in the dark for 14 days. Vegetative and reproductive structures were observed and measured (at least 30 measurements per structure type) from mounts prepared in Shear’s medium (3 g potassium acetate, 60 mL glycerol, 90 mL 95% ethanol, and 150 mL distilled water) [23] under an Olympus BH-2 bright-field microscope (Olympus Corporation, Tokyo, Japan). Photomicrographs were taken with a Zeiss Axio Imager M1 light microscope (Zeiss, Oberkochen, Germany) equipped with a DeltaPix InfinityX digital camera (DeltaPix, Smoerum, Denmark).

### 2.3. DNA Extraction, Amplification, and Sequencing

Total genomic DNA was extracted from colonies grown on PDA at 25 °C in the dark for 7–10 days following a modified protocol of Müller et al. [24] and quantified using a Nanodrop 2000 spectrophotometer (Thermo Scientific, Madrid, Spain). The set of molecular markers amplified for each fungal strain was selected based on the literature. The primer pairs ITS5/ITS4 [25] and LR0R/LR5 [26] were used to amplify the internal transcribed spacer (ITS) region and the D1–D2 domains of the 28S nrRNA (LSU), respectively. Additional markers included actin (*act*), fragments of the translation elongation factor 1α (*tef*-1α), and the RNA polymerase II subunits 1 and 2 (*rpb*1, *rpb*2), amplified with primers ACT-512F/ACT-783R [27], 983F/2218R [28], Bt2a/Bt2b [29], RPB2-5F2/fRPB2-7cR [30,31], and RPB1A-Ac/RPB1-Cr [32]. PCR products yielding a single band on agarose gels were stored at -20 °C and sequenced at Macrogen Europe (Macrogen Inc., Madrid, Spain) using the same primers. Consensus sequences were assembled using SeqMan v. 7.0.0 (DNAStar Lasergene, Madison, WI, USA).

### 2.4. Phylogenetic Analysis

To clarify the taxonomic placement of the studied strains and to evaluate their phylogenetic relationships within the corresponding lineages, a series of single-locus and multilocus phylogenetic analyses were conducted. Separate phylogenetic trees were inferred for each genus containing putatively novel taxa, using different combinations of molecular markers depending on data availability and their proven phylogenetic informativeness at the genus and family levels. Ribosomal DNA regions (ITS and LSU) were used as a backbone for all analyses due to their widespread use in fungal systematics and the availability of reference sequences, whereas protein-coding genes (*tef*-1α, *rpb*1 and *rpb*2) were incorporated when available to improve phylogenetic resolution and nodal support. In total, five independent phylogenetic reconstructions were performed, each corresponding to a different taxonomic group, allowing robust assessment of species boundaries and higher-level relationships.

Consensus sequences were compared against the National Center for Biotechnology Information (NCBI) database using the Basic Local Alignment Search Tool (BLAST+ version 2.17.0; https://blast.ncbi.nlm.nih.gov/Blast.cgi, accessed on 28 November 2025), and Mycobank Databases (https://www.mycobank.org/Pairwise_alignment, accessed on 28 November 2025) to obtain a preliminary molecular identification of the strains. A maximum level of identity (MLI) of ≥98% was considered sufficient for species-level identification, whereas lower values were interpreted as indicative of putative undescribed taxa [33,34]. Based on the BLAST results, single-locus and combined phylogenetic analyses were conducted for strains within each genus. Individual loci were aligned in MEGA v.7.0 [35] using the ClustalW algorithm [36], refined with MUSCLE [37], and manually adjusted when necessary. After confirming the absence of topological incongruence among single-locus datasets, alignments were concatenated into a single dataset. Phylogenetic reconstruction was performed using Maximum Likelihood (ML) and Bayesian Inference (BI). The ML analysis was carried out on the CIPRES Science Gateway [38] with RA × ML-HPC2 on XSEDE v.8.2.12 [39], applying the best-fit substitution model automatically selected by the portal. Node support was assessed with 1000 bootstrap pseudoreplicates, considering values ≥70% as significant [40]. The BI analysis was conducted with MrBayes v.3.2.6 [41], using the substitution model established by using jModelTest v.2.1.3 [38,42] under the Akaike Information Criterion. Analyses were run for 5 million MCMC generations with four chains (one cold and three heated), sampling every 1000 generations. The first 25% of sampled trees were discarded as burn-in, and posterior probabilities (pp) ≥ 0.95 were regarded as significant [43]. Phylogenetic trees were visualized with FigTree v.1.3.1 (http://tree.bio.ed.ac.uk/software/figtree/, accessed on 28 November 2025). Sequence alignments and phylogenetic trees were deposited in Zenodo (https://zenodo.org, accessed on 28 November 2025), whereas newly generated DNA sequences were deposited in GenBank (Table 1). Novel taxa were registered in MycoBank (https://www.mycobank.org/, accessed on 28 November 2025).

## 3. Results

As a result of our investigation, 49 strains of filamentous fungi were isolated. These strains, along with their identification based on phenotypic characterization and nucleotide sequence identity (>98%) of selected molecular markers and the accession numbers of the culture collections where the holotype and living strains have been deposited, are listed in Table A1.

After careful examination of the phenotypic features, as well as the sequence identity of selected molecular markers (Table 2), we concluded that certain strains of ours constituted putatively new species.

To ascertain that the strains listed in Table 2 represent species new to science, phylogenetic analyses based on single-locus and concatenated molecular markers (when possible) were conducted for each genus in which these strains were placed. Analyses of individual molecular markers for each genus revealed no topological incongruence among trees with ≥70% reciprocal bootstrap support, thereby validating the use of a combined multilocus approach.

**Figure 1:**
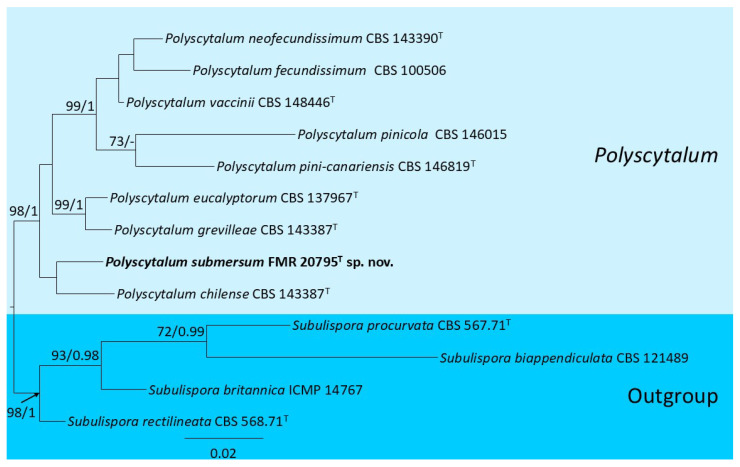
Maximum-likelihood analysis of the ITS  +  LSU concatenated alignment of strain FMR 20795 and eight species of *Polyscytalum*. RA × ML bootstrap support values (BS ≥ 70%) and Bayesian posterior probabilities (PP ≥ 0.95) are shown above the branches, and the novel species are shown in bold. The tree is rooted with *Subulispora biappendiculata* CBS 121489, *S. britannica* ICMP 14767, *S. procurvata* CBS 567.71, and *S. rectilineata* CBS 568.71. ^T^ = ex-type strain.

For *Polyscytalum* spp., the concatenated ITS  +  LSU dataset comprised nine ingroup strains (including our strain FMR 20795) and four outgroup strains (species of the genus *Subulispora*), yielding an alignment of 1425 characters (ITS = 586, LSU = 839). Of this total, 204 positions were variable (ITS = 116, LSU = 88), and 80 were parsimony informative (ITS = 65, LSU = 15). Maximum likelihood (ML) and Bayesian inference (BI) analyses produced congruent topologies. For ML, the best-fit substitution models were Tamura 3-parameter + γ (T92 + G) for ITS and Kimura 2-parameter + γ + I (K2 + G + I) for LSU; for BI, the preferred models were Hasegawa–Kishino–Yano + γ (HKY + G) for ITS and HKY variant + I (HK80 + I) for LSU. Support values exhibited only minimal differences between methods yet remained broadly concordant. The resultant phylogeny (Figure 1) resolved a strongly supported *Polyscytalum* spp. clade (98% BS; 1 PP), which bifurcates into two subclades. One contains most of the described species, while the other comprises the ex-type strain of *Polyscytalum chilense* (CBS 143387) and the strain FMR 20795. The genetic distance among these taxa is sufficient to consider FMR 20795 as a distinct species of *P. chilense*.

**Figure 2:**
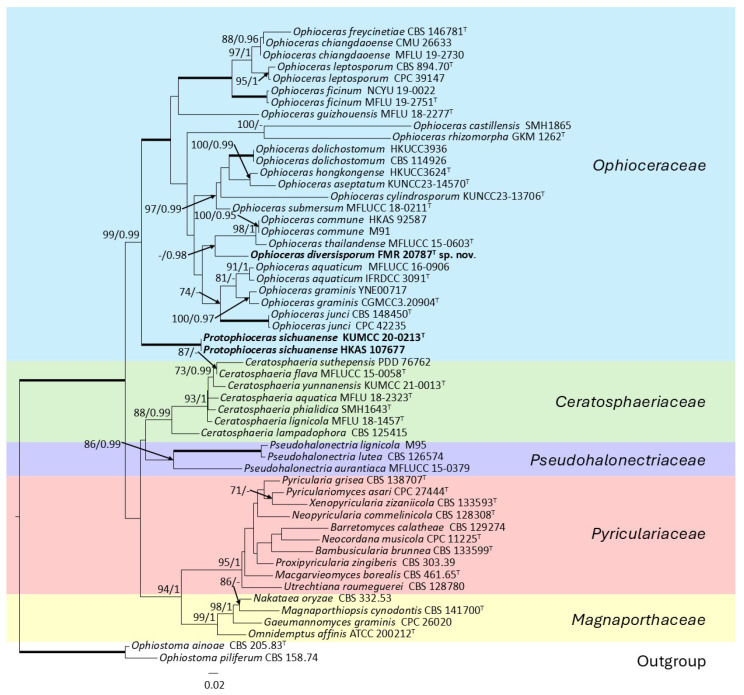
Maximum-likelihood analysis of the ITS  +  LSU  +  *rpb*1 concatenated nucleotide sequences of the *Magnaporthales*, comprising strain FMR 20787 and 52 representative species of the order. RA × ML bootstrap support values (BS ≥ 70%) and Bayesian posterior probabilities (PP ≥ 0.95) are shown above the branches. Fully supported branches (100% BS/1 PP) are represented as thick lines. Novel species and combinations are shown in bold. The tree was rooted using *Ophiostoma ainoae* CBS 205.83 and *O. piliferum* CBS 158.74. ^T^ = ex-type strain.

The phylogenetic inference for *Ophioceras* spp., as well as for other members of the order *Magnaporthales*, was built based on a concatenated ITS + LSU + *rpb*1 nucleotide alignment comprising 52 ingroup strains, comprising our strain, FMR 20787, and two outgroup strains of the genus *Ophiostoma*. The final dataset encompassed 2597 characters (including gaps): 729 bp for ITS, 869 bp for LSU, and 999 bp for *rpb*1. Among these positions, 1235 were variable (ITS = 412; LSU = 251; *rpb*1 = 572) and 939 were parsimony informative (ITS = 297; LSU = 199; *rpb*1 = 443). Maximum likelihood (ML) and Bayesian inference (BI) analyses yielded congruent topologies, indicating methodological consistency. For the ML analysis, the best-fit substitution models were K2 + G + I for ITS, K2 + G for LSU, and TN93 + G for *rpb1*. In the BI framework, the general GTR + G + I model was selected for all three molecular markers. Phylogenetic reconstruction (Figure 2) resolved two major clades, one exclusively comprising the family *Ophioceraceae* and the other encompassing the remaining *Magnaporthales*, each further subdivided into two strongly supported subclades. Within *Ophioceraceae*, all known species of *Ophioceras* form a monophyletic lineage, with the sole exception of *O. sichuanense*, which emerges as a fully supported, deeply divergent branch. Its pronounced phylogenetic distinctness and basal position justify its recognition as a new genus. Likewise, the strain FMR 20787 occupies a unique terminal branch within the core *Ophioceras* clade, sister to *O. commune* and *O. thailandense*, justifying its description as a novel species.

**Figure 3:**
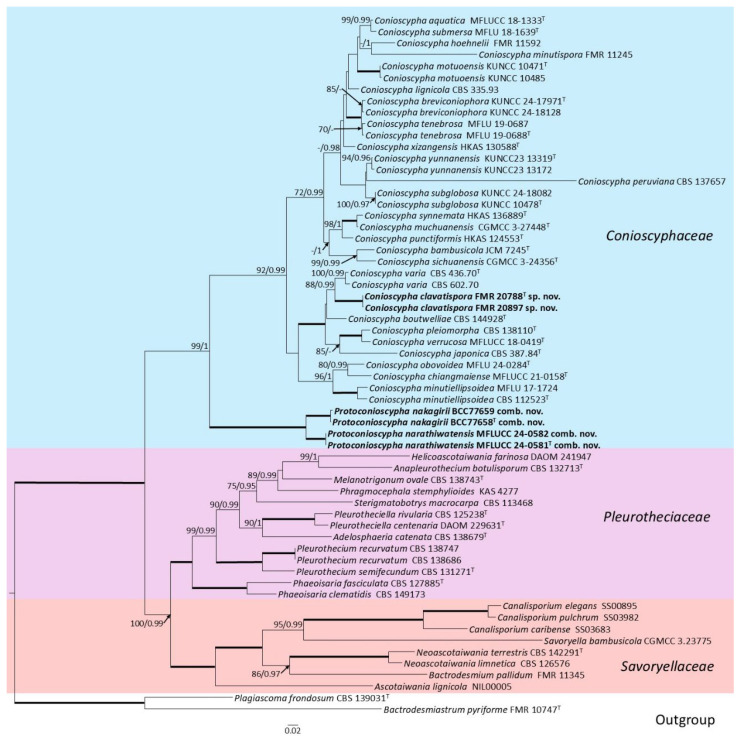
Maximum-likelihood analysis of the ITS  +  LSU  +  *rpb*2 concatenated nucleotide sequences of 59 representative taxa of the families *Conioscyphaceae*, *Pleurotheciaceae*, and *Savoryellaceae*, together with strains FMR 20788 and FMR 20897 and two outgroup taxa. RAxML bootstrap support values (BS ≥ 70%) and Bayesian posterior probabilities (PP ≥ 0.95) are shown above the branches. Fully supported branches (100% BS/1 PP) are represented as thick lines. Novel species and combinations are shown in bold. The tree was rooted using *Bactrodesmiastrum pyriforme* FMR 10747 and *Plagiascoma frondosum* CBS 139031. ^T^ = ex-type strain.

Phylogenetic analysis of the families *Conioscyphaceae*, *Pleurotheciaceae*, and *Savoryellaceae* was conducted on a concatenated ITS–LSU–*rpb*2 dataset comprising 59 ingroup strains, containing the strains FMR 20788 and FMR 20897, and two outgroups (*Bactrodesmiastrum pyriforme* and *Plagiascoma frondosum*). The total length of the final alignment was 2680 characters (including gaps: 864 ITS, 860 LSU, 956 *rpb*2), of which 1224 were parsimony informative (545 ITS, 262 LSU, 417 *rpb*2) and 1511 were variable sites (660 ITS, 367 LSU, 484 *rpb*2). Maximum likelihood (ML) and Bayesian inference (BI) analyses produced congruent topologies. For ML, the best-fit substitution models were T92 + G (ITS), TN93 + G (LSU) and T92 + G + I (*rpb2*); in BI, a unified GTR + I + G model was applied to all three phylogenetic markers. Phylogenetic reconstruction (Figure 3) resolved two principal clades, one exclusively comprising the family *Conioscyphaceae* and the other encompassing the families *Pleurotheciaceae* and *Savoryellaceae*, and further subdividing the *Conioscyphaceae* into two strongly supported subclades: one containing the bulk of *Conioscypha* species, in which the strains FMR 20788 and FMR 20897 form a fully supported terminal branch sister to *C. varia*, and the other harboring exclusively *C. nakagirii* and *C. narathiwatensis* on its own distinct, fully supported branch. Given the pronounced phylogenetic divergence and discrete placements of FMR 20788 and FMR 20897 and of *C. nakagirii* and *C. narathiwatensis*, they warrant formal recognition as a novel species and as a novel genus, respectively.

**Figure 4:**
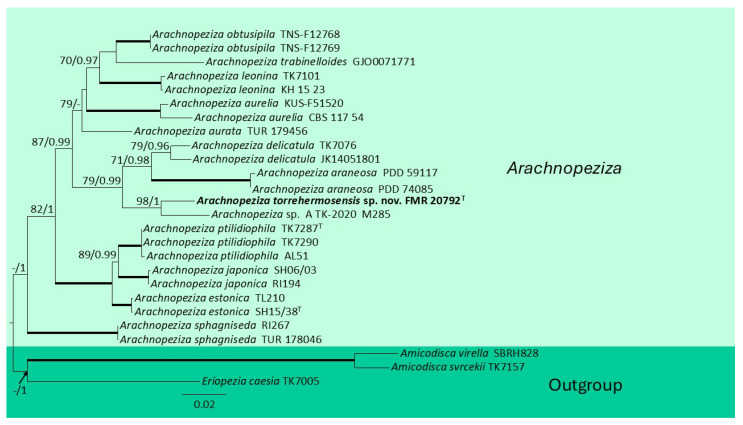
Maximum-likelihood analysis of the ITS  +  LSU  +  *tef*-1α  +  *rpb*1 concatenated nucleotide sequences of species of the genus *Arachnopeziza*, including our strain *Arachnopeziza torrehermosensis* FMR 20792^T^. RA × ML bootstrap support values (BS ≥ 70%) and Bayesian posterior probabilities (PP ≥ 0.95) are shown above the branches. Fully supported branches (100% BS/1 PP) are represented as thick lines. New species are shown in bold. The tree was rooted using *Amicodisca svrcekii* TK7157, *A. virella* SBRH828, and *Eriopezia caesia* TK7005. ^T^ = ex-type strain.

For the phylogenetic inference of *Arachnopeziza* spp., the ultimate concatenated ITS + LSU+ *tef-*1α +*rpb*1 dataset encompassed 23 ingroup strains, including the strain FMR 20792, and three outgroup strains (of the genera *Amicodisca* and *Eriopezia*). The alignment length reached 2532 characters including gaps (511 for ITS, 548 for LSU, 732 for *tef-*1α and 741 for *rpb*1), 457 of them being parsimony informative (120 for ITS, 44 for LSU, 94 for *tef-*1α and 199 for *rpb*1) and 592 of them variable sites (146 for ITS, 73 for LSU, 140 for *tef-*1α and 233 for *rpb*1). The similar topologies and scarce differences in both the ML and the BI analysis indicated that they were congruent. The best fitting model for each molecular marker in the ML analysis was K2 + G for ITS, *tef-*1α, and *rpb1* and JC for LSU. The best fitting model for each molecular marker in the BI analysis was SYM + G for ITS and *tef-*1α, K80 + G for LSU and *rpb*1. The phylogenetic analysis (Figure 4) revealed that our strain FMR 20792 was placed in an independent, well-supported branch within the phylogenetic tree, close to *A. delicatula*, but at a sufficient genetic distance to be considered a distinct species; thus, FMR 20792 represents a novel species of *Arachnopeziza*.

**Figure 5:**
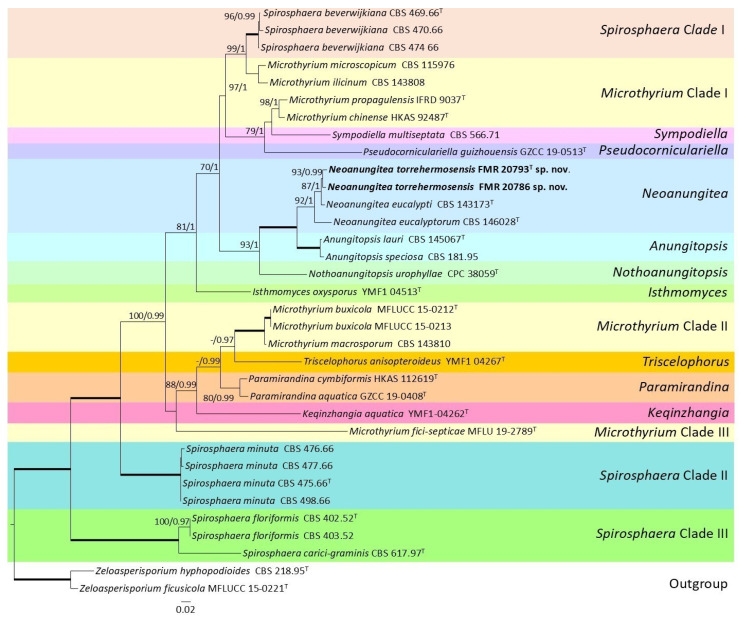
Phylogenetic analysis of members of the family *Microthyriaceae* obtained from the combined ITS and LSU nucleotide sequences of 32 representative taxa of the family, including strains FMR 20793 and FMR 20786. RA × ML bootstrap support values (BS ≥ 70%) and Bayesian posterior probabilities (PP ≥ 0.95) are shown above the branches. Fully supported branches (100% BS/1 PP) are represented as thick lines. New species are shown in bold. The tree was rooted with *Zeloasperisporium ficusicola* MFLUCC 15-0221 and *Zeloasperisporium hyphopodioides* CBS 218.95. ^T^ = ex-type strain.

Regarding the phylogenetic analysis of members of the *Microthyriaceae*, whose taxa were molecularly the closest to our strains FMR 20793 and FMR 20786, the concatenated ITS + LSU dataset contained 32 ingroup strains (including our strains) and two outgroup strains of the genus *Zeloasperisporium*. The length of the alignment, including gaps, was 1549 characters (594 for ITS and 955 for LSU). Among the total characters of the alignment, 636 of them were parsimony informative (358 for ITS and 278 for LSU) and 791 of them were variable sites (429 for ITS and 362 for LSU). The ML and BI analysis were both considered congruent because they displayed little to no differences. The model that fitted the best for every molecular marker in the ML analysis was K2 + G for ITS and TN93 + G for LSU. The model that fitted the best for every molecular marker in the BI analysis was HKY + I + G for ITS and GTR + I + G for LSU. The phylogenetic tree (Figure 5) displayed sixteen terminal clades within the *Microthyriaceae*, revealing the polyphyletic nature of the genera *Microthyrium* and *Spirosphaera*. Our strains FMR 20793 and FMR 20786 clustered within a well-supported terminal branch comprising species of *Neoanungitea*, closely related to *Neoanungitea eucalypti*. This branch is part of a broader, well-supported terminal clade that also includes species of *Anungitopsis*, with *Nothoanungitopsis urophyllae* occupying a basal position.

### Taxonomy

*Xylariales* Nannf., Nova Acta R. Soc. Scient. upsal., Ser. 4 8 (no. 2): 66 (1932). Mycobank MB 90505.

*Polyscytalum* Riess, Bot. Ztg. 11: 138 (1853). Mycobank MB 9508.

**Figure 6:**
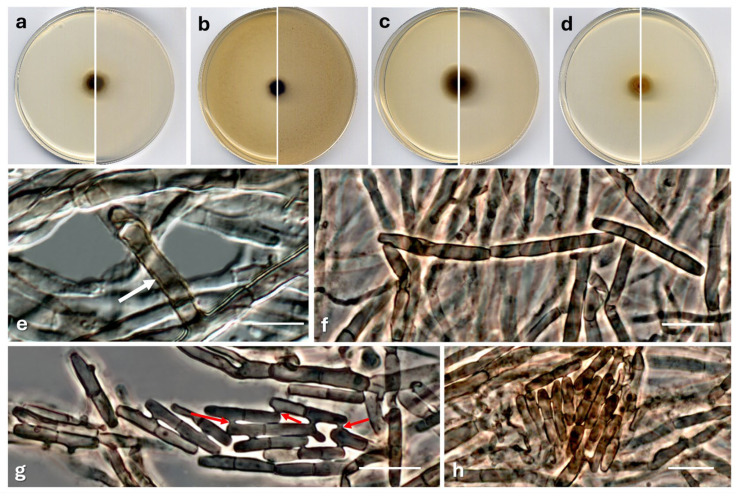
*Polyscytalum submersum* CBS 154003^T^. Colonies on PCA (**a**), OA (**b**), MEA (**c**), and PDA (**d**) after two weeks at 25 °C ((**left**), surface; (**right**), reverse). Conidiophore (white arrow) (**e**). Conidia in chains (**f**). Anastomosing conidia (red arrows, anastomosis tubes) (**g**,**h**). Scale bars (**e**–**h**) = 10 µm.

*Polyscytalum submersum* Barnés-Guirado, Cano & Stchigel, sp. nov. MycoBank MB 859383. Figure 6.

Etymology. From Latin *submersum*, submerged, because the fungus was isolated from plant debris submerged into freshwater.

Description: *Hyphae* septate, hyaline to pale brown, smooth- and thick-walled, branching, 1.0–3.0 µm wide. *Conidiophores* semi-micronematous, solitary, erect, straight to flexuous, 1–5-septate, unbranched, pale brown, smooth- and thin-walled, cylindrical to subcylindrical, 7.0–35.0 × 2.0–3.0 µm. *Conidiogenous* cells terminal, mostly integrated, sometimes discrete, pale brown, smooth-walled, cylindrical, subcylindrical or irregularly shaped, 4.0–15.0 × 2.0–3.0 µm, proliferating sympodially, with flat-tipped scars. *Conidia* holoblastic, 1–3-septate, pale brown, smooth- and thin-walled, disposed in chains of up to 5 conidia, cylindrical with truncated ends, 8.0–23.0 × 2.0–3.0 µm, non-guttulate, linked among them through anastomosis tubes.

Culture characteristics (after 14 d at 25 °C)—Colonies on PDA 15 mm diam., raised at the center, flattened at the edges, velvety, wrinkled at the center, smooth at the margins, golden yellow (5B6) to brownish yellow (5C7) at the center, white (1A1) at the margins, lobulated, filamentous margins, sporulation absent; reverse yellowish brown (5C5) to yellowish brown (5C8) at the center, white (1A1) at the edges, soluble pigment absent. Colonies on PCA reaching 17 mm diam., circular, slightly raised at the center, flattened at the margins, cottony to velvety, radially sulcate, dark brown (6F7) to brown (6E7) at the center, white (1A1) towards the irregular margins, sporulation absent; reverse dark brown (6F8) at the center, white (1A1) at the margins; soluble pigment absent. Colonies on OA reaching 16 mm diam., circular, flattened, velvety to powdery, smooth margins, olive brown (4F7) at the center, white (1A1) at the filamentous margins, sporulation absent; reverse olive brown (4F8) at the center, white (1A1) at the margins; soluble pigment absent. Colonies on MEA reaching 24 mm diam., circular, slightly raised at the center, flattened at the margins, felty, radially sulcate, brown (5F8) at the center, grayish brown (5D3) at the filamentous margins, sporulation absent; reverse brown (5F8) at the center, yellowish brown (5E4) to white (1A1) at the margins; soluble pigment absent. Cardinal temperatures of growth: minimum 5 °C, optimum 25 °C, and maximum 30 °C.

Typus. SPAIN, Extremadura community, Badajoz province, Zújar River (38°24′31.4″ N 5°34′47.9″ W) (Granja de Torrehermosa), isolated from submerged decomposing unidentified leaf, 12 November 2022, collected by Juan R. García Martínez, isolated by María Barnés Guirado (holotype CBS H-25763; cultures ex-type CBS 154003 = FMR 20795).

Notes: The genus *Polyscytalum* is characterized by producing hyaline to pale brown and smooth-walled, septate, cylindrical conidia with truncated ends, features observed in the new species, *Polyscytalum submersum*. Morphologically, *P. submersum* differs from its closest species, *P. chilense*, by having 1–5-septate conidiophores (1–3-septate in *P. chilense*), and non-guttulate and anastomosing conidia (guttulate and non-anastomosing in *P. chilense*).

*Magnaporthales* Thongk., Vijaykr. & K.D. Hyde, Fungal Diversity 34: 168 (2009).

*Ophioceraceae* Klaubauf, E.G. LeBrun & Crous, Stud. Mycol. 79: 103 (2014). Mycobank MB 810201.

*Ophioceras* Sacc., Syll. fung. (Abellini) 2: 358 (1883). Mycobank MB 3595.

**Figure 7:**
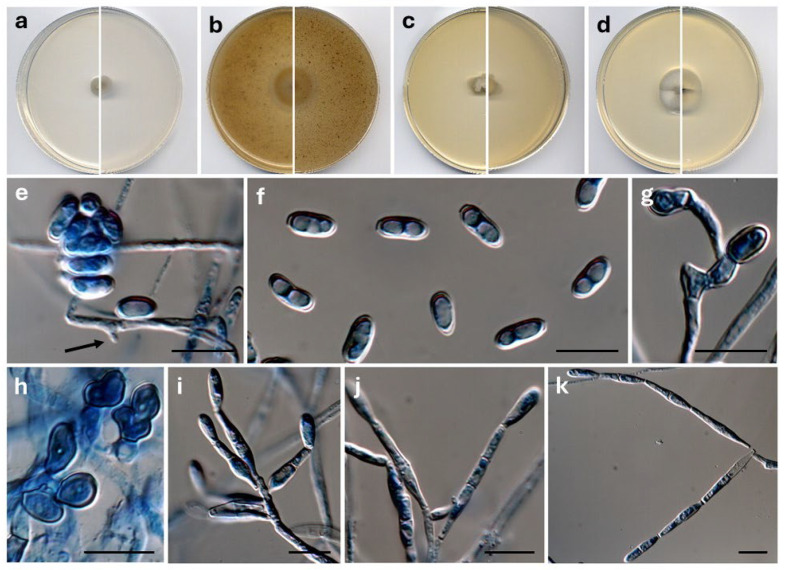
*Ophioceras diversisporum* CBS 154004^T^. Colonies on PCA (**a**), OA (**b**), MEA (**c**) and PDA (**d**) after two weeks at 25 °C ((**left**), surface; (**right**), reverse). Conidia grouped in a mucous mass. Down, an adelophialide (black arrow) (**e**). Free conidia (**f**). Appressoria (**g**,**h**). Synanamorph: conidiophores producing chains of conidia (**i**–**k**). Scale bars (**e**–**k**) = 10 µm.

*Ophioceras diversisporum* Barnés-Guirado, Stchigel & Cano, sp. nov. MycoBank MB 859384. Figure 7.

Etymology. From Latin *diversus*-, different, and -*sporae*, spores, because the fungus produces two types of conidia.

Description: *Hyphae* hyaline to subhyaline, septate, smooth- and thick-walled, branched, 1.0–2.0 µm wide. *Asexual morph*—*Conidiophores* semi-micronematous to micronematous, reduced to the conidiogenous cells. *Conidiogenous* cells phialidic, terminal or lateral, hyaline, smooth- and thin-walled, cylindrical to flask-shaped, 8.0–17.0 × 1.0–2.0 µm, conical when integrated (adelophialides) and measuring 1.0–2.0 × 1.0 µm, producing conidia in mucous masses. *Conidia* unicellular, enteroblastic, solitary, hyaline, smooth- and thick-walled, 2–3-guttulate, ellipsoidal to slightly reniform, 7.0–10.0 × 3.0–4.5 µm. *Synanamorph*—*Conidiophores* micronematous, reduced to the conidiogenous cells. *Conidiogenous* cells uni- or polyblastic, lateral or terminal, integrated and indistinguishable from the vegetative hyphae. *Conidia* holoblastic, 0–2-septate, mostly fusiform to navicular, sometimes clavate (when terminal), flattened at the base or both ends, 11.0–30.0 × 2.0–4.0 µm, guttulate, disposed in straight or single branching acropetal chains of up to 8 conidia. *Appressoria* abundant, single, dark brown, lateral or terminal on the vegetative hyphae mameliform, pyriform or irregularly shaped and with lobulated margins, 4.0–14.0 × 2.5–8.5 µm, flattened at the base and with a lateral or terminal germ pore; in this later case, a new hypha protrudes through the pore and eventually generates a new terminal appressorium, which can eventually proliferate again.

Culture characteristics (after 14 d at 25 °C)—Colonies on PDA reaching 24 mm diam., circular, umbonate, velvety, slightly wrinkled, pale gray (1B1) at the center and white (1A) at the margins, margins entire, sporulation abundant; reverse white (1A1); soluble pigment absent. Colonies on PCA reaching 12 mm diam., circular, slightly raised at the center, flattened at the margins, velvety to powdery, white (1A1), entire, sporulation scarce; reverse yellowish brown (5D8) at the center, white (1A1) at the margins; soluble pigment absent. Colonies on OA reaching 28 mm diam., circular, flattened, velvety to powdery, pale yellow (4A4) to grayish yellow (4B5) at the center, white (1A1) at the margins, entire, sporulation moderate to abundant; reverse grayish yellow (4B6) to light (4A4) at the center, grayish yellow (4C6) to white (1A1) towards margins; soluble pigment absent. Colonies on MEA reaching 15 mm diam., lobulated, irregular and spreading, slightly raised at the center, flattened at the margins, velvety to powdery, white (1A1), sporulation absent; reverse yellowish brown (5E8) at the center, white (1A1) at the edges; soluble pigment absent. Cardinal temperatures of growth: minimum 15 °C, optimum 25 °C, and maximum 30 °C.

Typus. SPAIN, Extremadura community, Badajoz province, Zújar River (38°24′31.4″ N 5°34′47.9″ W) (Granja de Torrehermosa), isolated from submerged decomposing unidentified twig, 12 November 2022, collected by Juan R. García Martínez, isolated by María Barnés Guirado (holotype CBS H-25764; cultures ex-type CBS 154004 = FMR 20787).

Notes: The genus *Ophioceras* is characterized by the production of immersed, globose, dark, ostiolate perithecia; cylindrical, elongated asci; and typically filiform, septate ascospores. Most species do not produce an asexual morph, with the exceptions of *O. graminis*, which also lacks a sexual morph, and *O. rhizomorphum*. Although *O. diversisporum* is phylogenetically closely related to *O. commune*, it only produces an asexual morph, like *O. graminis*. However, *O. diversisporum* differs morphologically from *O. graminis* by producing ellipsoidal to slightly reniform, 2–3-guttulate conidia, in contrast to the lunate, allantoid to fusiform conidia of *O. graminis*. Additionally, *O. diversisporum* produces a synanamorph, a feature not reported in *O. graminis*, as well as appressoria, whereas *O. graminis* forms hyphopodia. In contrast, *O. rhizomorpha* produces a didymobotryum-like asexual morph, characterized by synnemata bearing an apical fertile region with tretic conidiophores. This morphology is distinct from the known anamorphs of *O. graminis* and *O. diversisporum*.

*Protophioceras* Barnés-Guirado, Cano & Stchigel, gen. nov. MycoBank MB 859580.

Etymology. From Latin *protos*- meaning ‘first’, referring to the fungus’ phylogenetic relationship, as it occupies a basal position relative to the species of *Ophioceras*.

Description: *Pseudostromata* carbonaceous, scattered, solitary, semi-immersed to erumpent, 1–5-loculate, glabrous, ostiolate, papillate; locules immersed within the pseudostroma, clustered, subglobose to ampulliform, with a long black periphysate neck; *peridium* of *textura angularis* to *textura prismatica*, thick-walled, composed of several layers of broad and flattened pseudoparenchymatous cells; *paraphyses* present; *asci* 8-spored, unitunicate, cylindrical, sessile to subsessile, with a bulbous-like base and a J-shaped apical ring; *ascospores* hyaline, parallelly disposed or overlapping, filiform to sigmoidal, aseptate, thin- and smooth-walled, multi-guttulate. *Asexual morph* not observed.

Type species: *Protophioceras sichuanense* (Jiang, Phookamsak & Hyde) Barnés-Guirado, Cano & Stchigel, comb. nov. MycoBank MB 859589.

Description: H.B. Jiang, Phookamsak and K.D. Hyde (2021) [44].

Notes: Despite the overall similarity in ascus and ascospore morphology between *Protophioceras sichuanense* and species of *Ophioceras*, the two taxa differ markedly in their ascomatal architecture. *Protophioceras sichuanense* is characterized by the formation of well-developed, polyloculate pseudostromata bearing multiple ostiolate necks, a feature not observed in *Ophioceras*. In contrast, species of *Ophioceras* consistently produce solitary, uniloculate perithecial ascomata with a single neck, lacking any stromatic tissue. This fundamental difference in ascomatal organization reflects distinct developmental patterns and has traditionally been regarded as taxonomically informative at the generic level within the Magnaporthales. Moreover, the separation of *P. sichuanense* from *Ophioceras* is strongly supported by multilocus phylogenetic analyses, in which *P. sichuanense* forms a deeply divergent, fully supported lineage basal to the core *Ophioceras* clade. The congruence between these pronounced morphological differences and the phylogenetic evidence supports the recognition of *Protophioceras* as a distinct genus.

*Conioscyphales* Réblová & Seifert, in Réblová, Seifert, Fournier & Štěpánek, Persoonia 37: 63 (2016). MycoBank MB 813226.

*Conioscyphaceae* Réblová & Seifert, in Réblová, Seifert, Fournier & Štěpánek, Persoonia 37: 63 (2016). MycoBank MB 813227.

*Conioscypha* Höhn., Annls mycol. 2 (1): 58 (1904). MycoBank MB 7754.

**Figure 8:**
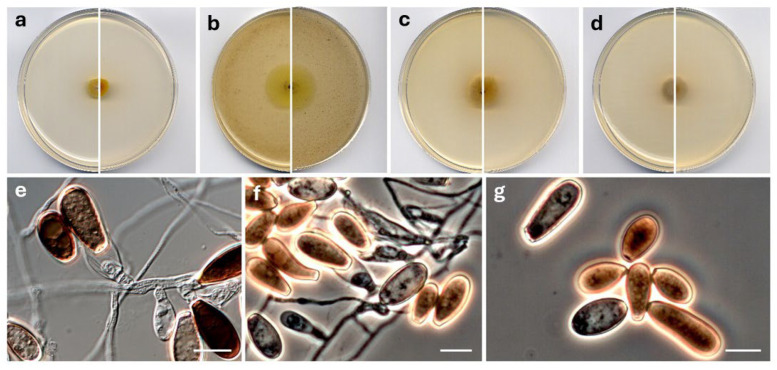
*Conioscypha clavatispora* CBS 154005^T^. Colonies on PCA (**a**), OA (**b**), MEA (**c**) and PDA (**d**) after two weeks at 25 °C ((**left**), surface; (**right**), reverse). Conidiogenous cells and conidia (**e**,**f**). Free conidia (**g**). Scale bars ((**e**–**g**) = 10 µm).

*Conioscypha clavatispora* Barnés-Guirado, Cano & Stchigel, sp. nov. MycoBank MB 859385. Figure 8.

Etymology. From Latin *clavatum*-, clavate, and -*sporae*, spores, due to the morphology of the conidia.

Description: *Hyphae* mostly immersed, septate, hyaline, branched, smooth- and thin-walled, 0.5–1.0 μm wide. *Conidiophores* micronematous, mononematous, reduced to conidiogenous cells. *Conidiogenous* cells monoblastic, discrete, sessile, arising terminally or laterally on the hyphae, solitary or in small clusters, hyaline, smooth- and thin-walled, cupulate, 14.0–30.0 μm long, 5.0–12.0 μm in the widest part, with a multi-layered cupulate collarette after two percurrent elongations. *Conidia* partially holoblastic, unicellular, solitary, endogenous, pale brown to brown, smooth-walled to asperulate, thick-walled, ellipsoidal to clavate, 9.5–22.0 × 4.5–10.0 μm, truncated at the base and with a median pore, rounded at the top, non-guttulate or mono- to multi-guttulate.

Culture characteristics (after 14 d at 25 °C)—Colonies on PDA reaching 14 mm diam., flat, round, velvety, margins radiate and filamentous, reddish yellow (4A6 to 4A7), sporulation absent; reverse yellowish orange (4B7), dark yellow (4C8) at the center; soluble pigment absent. Colonies on PCA reaching 12 mm diam., cottony to velvety, slightly raised at the center, margins filamentous and slightly lobulated, reddish yellow (4A7), sporulation moderate to abundant, reverse deep orange (5A8), golden yellow (5B7) at the center; soluble pigment absent. Colonies on OA reaching 27 mm diam., flat, velvety, margins filamentous, pale yellow (3A5), grayish yellow (3B7) at the center, sporulation abundant; reverse yellow (3A6), grayish yellow (3B7) at the center; soluble pigment absent. Colonies on MEA reaching 17 mm diam., slightly raised at the center, cottony to velvety, margins filamentous and slightly lobulated, reddish yellow (4A6), sporulation abundant; reverse deep yellow (4A8); soluble pigment absent. Cardinal temperatures of growth: minimum 15 °C, optimum 25 °C, and maximum 37 °C.

Typus. SPAIN, Extremadura community, Badajoz province, Zújar River (38°24′31.4″ N 5°34′47.9″ W) (Granja de Torrehermosa), isolated from submerged plant debris, 12/11/2022, collected by Juan R. García Martínez, isolated by María Barnés Guirado (holotype CBS H-25765; cultures ex-type CBS 154005 = FMR 20788).

Other specimens. SPAIN, Extremadura community, Badajoz province, Zújar River (38°24′31.4″ N 5°34′47.9″ W) (Granja de Torrehermosa), isolated from submerged decomposing unidentified twig, 12 November 2022, collected by Juan R. García Martínez, isolated by María Barnés Guirado (living culture FMR 20897).

Notes: While *Conioscypha clavatispora* shares with other species in the genus the morphology of the conidiophores, conidiogenous cells, and conidiogenesis, it differs from its closest relative, *Conioscypha varia*, in several key features. The conidia of *C. clavatispora* are ellipsoidal to clavate, whereas those of *C. varia* are ovoid, flamiform, navicular, or subellipsoidal. Additionally, the conidia of *C. clavatispora* (9.5–22 × 4.5–10 μm) are larger than those of *C. varia* (8.4–15 × 5.6–8.5 μm). The conidial apex in *C. clavatispora* is rounded with a truncated base, while in *C. varia* the apex may be rounded or pointed, and the base may be rounded or truncated. These species also differ in conidial wall ornamentation: conidia in *C. clavatispora* are smooth to asperulate, while those in *C. varia* are smooth. Furthermore, conidia of *C. clavatispora* typically exhibits a median pore and guttules, which are absent in *C. varia*.

*Protoconioscypha* Barnés-Guirado, Cano & Stchigel, gen. nov. MycoBank MB859603.

Etymology. From Latin *protos*- meaning ‘first’, referring to the fungus’s phylogenetic relationship, as it occupies a basal position relative to the species of *Conioscypha*.

Description: *Hyphae* superficial to immersed, branched, smooth-walled, septate, brown, pale brown or hyaline. *Conidiophores* micronematous to semi-macronematous, mononematous, erect, arising terminally or laterally on the hyphae, solitary or in small clusters. *Conidiogenous cells* monoblastic, integrate or discrete, sessile or over small conidiophores, cuneiform, cylindrical, smooth-walled, percurrently proliferating, from multiple or a single cup-shaped collarettes up to 50 mm wide at the apex to no collarettes. *Conidia* solitary, turbinate to pyriform, smooth-walled, unicellular, aseptate, rounded at apex, rounded to truncate with a pore to no pore at the base. *Sexual morph* not observed.

Type species: *Protoconioscypha nakagirii* (Chuaseeharonnachai, Somrithipol, Suetrong & Boonyuen) Barnés-Guirado, Cano & Stchigel, comb. nov. MycoBank MB 859605.

Description: Chuaseeharonnachai, Somrithipol, Suetrong and Boonyuen (2016) [45].

Other species: *Protoconioscypha narathiwatensis* (Karimi, Asghari & Hyde) Barnés-Guirado, Cano & Stchigel, comb. nov. MycoBank MB861481.

Description: Karimi, Asghari and Hyde (2025) [46].

Notes: Although there are several morphological similarities between *Protoconioscypha nakagirii*, *Protoconioscypha narathiwatensis* and the species of the genus *Conioscypha*, the formers produce turbinate to pyriform conidia, feature not observed in any of the species of *Conioscypha*. Moreover, the conidia in *P. nakagirii* and *P. narathiwatensis* are larger than any conidia produced by a *Conioscypha* spp.

*Helotiales* Nannf., Nova Acta R. Soc. Scient. upsal., Ser. 4 8 (no. 2): 68 (1932). MycoBank MB 90476.

*Arachnopezizaceae* Hosoya, J.G. Han & Baral, in Baral, Index Fungorum 225: 1 (2015). MycoBank MB 551075.

*Arachnopeziza* Fuckel, Jb. nassau. Ver. Naturk. 23–24: 303 (1870). MycoBank MB 294.

*Arachnopeziza torrehermosensis* Barnés-Guirado, Stchigel & Cano, sp. nov. MycoBank MB 859386.

Etymology. From the name of the municipal district where the samples from the Zújar River were collected, “Granja de Torrehermosa”.

Description: *Mycelium* composed of septate, hyaline, refringent, smooth- and thin-walled, sinuous to loosely coiled, branching hyphae of 1.0–3.0 (–4.0) μm wide, with mostly thickened septa, occasionally narrowing at the level of the septum into a series of consecutive cells, sometimes incrusted of uncolored crystals. *Asexual or sexual morphs* not produced in all culture media tested after two months.

Culture characteristics (after 14 d at 25 °C)—Colonies on PDA reaching 25 mm diam., umbonate, velvety, wrinkled at the center, smooth at the margins, light gray (1D1) to white (1A1) at the center, white (1A1) at the margins, filamentous margins, sporulation absent; reverse yellowish brown (5F8) to golden brown (5D7) at the center, white (1A1) at the margins; soluble pigment absent. Colonies on PCA reaching 18 mm diam., slightly raised at the center, flattened at the margins, velvety, smooth, yellow (5C8) at the center, light orange (5A4) towards the margins, entire, sporulation absent; reverse orange (5B8) at the center, light orange (5A5) at the margins; soluble pigment absent. Colonies on OA reaching 9 mm diam., slightly raised at the center, flattened at the margins, velvety, smooth, filamentous margins, white (1A1) at the center, grayish yellow (4B4) at the margins, sporulation absent; reverse reddish yellow (4A6) at the center, pale yellow (4A3) at the margins; soluble pigment absent. Colonies on MEA reaching 25 mm diam., slightly raised at the center, flattened at the margins, cottony at the center, velvety at the margins, smooth, filamentous margins, white (1A1) at the center, grayish yellow (4B4) at the margins, sporulation absent; reverse dark yellow (4C8) at the center, grayish yellow (4B4) towards margins; soluble pigment absent. Cardinal temperatures of growth: minimum 5 °C, optimum 25 °C, and maximum 30 °C.

Typus. SPAIN, Extremadura community, Badajoz province, Zújar River (38°24′31.4″ N 5°34′47.9″ W) (Granja de Torrehermosa), isolated from submerged decomposing unidentified leaf, 12 November 2022, collected by Juan R. García Martínez, isolated by María Barnés Guirado (holotype CBS H-25766; cultures ex-type FMR 20792).

Notes: Although our efforts to induce the production of reproductive structures by the strain FMR 20792 were unsuccessful, the phylogenetic distance and placement respect to the rest of the species of that genus are consistent enough to consider *Arachnopeziza torrehermosensis* as a novel species.

*Microthyriales* G. Arnaud, Les Astérinées: 85 (1918). MycoBank MB 90485.

*Microthyriaceae* Sacc., Syll. fung. (Abellini) 2: 658 (1883). MycoBank MB 81008.

*Neoanungitea* Crous, in Crous et al., Persoonia 39: 359 (2017). MycoBank MB 823489.

**Figure 9:**
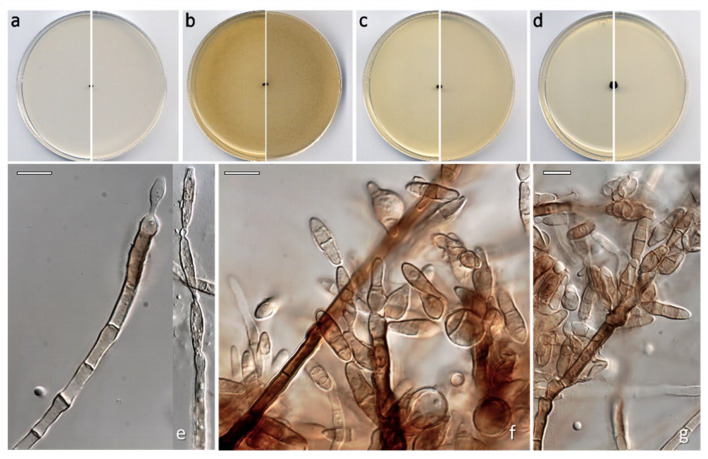
*Neoanungitea torrehermosensis* CBS 154006^T^. Colonies on PCA (**a**), OA (**b**), MEA (**c**) and PDA (**d**) after two weeks at 25 °C ((**left**), surface; (**right**), reverse). Young (**e**) and mature (**f**,**g**) conidiophores and conidia. Scale bars ((**e**–**g**) = 10 µm).

*Neoanungitea torrehermosensis* Barnés-Guirado, Stchigel & Cano, sp. nov. MycoBank MB 859387. Figure 9.

Etymology. From the name of the municipal district where the samples from the Zújar River were collected, “Granja de Torrehermosa”.

Description: *Hyphae* brown, septate, slightly verrucose, thick-walled, branching, 2–3 µm wide. *Conidiophores* macronematous, erect, dark brown, (1–)2–5-septate, straight at the base, slightly flexuous at the upper part, smooth- and thick-walled, subcylindrical, 60–250 × 4–8 µm. *Conidiogenous* cells sympodially proliferating, hyaline to dark brown, terminal or subterminal, in the latter case due to the percurrent proliferation of the conidiophore, cylindrical, barrel-shaped to ellipsoid with a truncate base, 21–37 × 3–6 µm, sometimes geniculate at the apex, bearing 1–7 not darkened nor thickened inconspicuous scars. *Ramoconidia* scarce, 0–3-septate, pale brown to brown, in acropetal chains of up to 3 conidia, smooth-walled to slightly verrucose, thin- to moderately thick-walled, cylindric, ellipsoidal or irregularly shaped, 17.5–32 × 3–5 µm, with a basal scar and lateral and apical scars, sometimes proliferating sympodially. *Conidia* holoblastic, (0) 1–3-septated, rarely with one oblique septum, in acropetal chains of up to 4 conidia, pale brown to brown, cylindric, fusiform, navicular, rarely pyriform to limoniform, 8.5–31 × 3–13 µm, truncated at the base (1–2 µm diam.) or (rarely) at both ends, not constricted or slightly constricted at the septum.

Culture characteristics (after 14 d at 25 °C)—Colonies on PDA reaching 5 mm diam., slightly raised at the center, flattened at the margins, velvety, smooth, dark brown (6F7), filamentous and irregular margins, sporulation moderate to abundant; reverse dark brown (6F8), soluble pigment absent. Colonies on PCA reaching 2 mm diam., raised, velvety, smooth, dark brown (6F7), filamentous margins, moderate to abundant sporulation; reverse dark brown (6F8), soluble pigment absent. Colonies on OA reaching 4 mm diam., raised, velvety, wrinkled, dark brown (6F6), slightly irregular margins, abundant sporulation; reverse dark brown (6F7), soluble pigment absent. Colonies on MEA reaching 4 mm diam., raised at the center, flattened at the edges, velvety, wrinkled, olive brown (4F7), irregular margins, abundant sporulation; reverse olive brown (4F8), soluble pigment absent. Cardinal temperatures of growth: minimum 15 °C, optimum 25 °C, and maximum 30 °C.

Specimen: SPAIN, Extremadura community, Badajoz province, Zújar River (38°24′31.4″ N 5°34′47.9″ W) (Granja de Torrehermosa), isolated from submerged decomposing unidentified leaf, 12 November 2022, collected by Juan R. García Martínez, isolated by María Barnés Guirado (holotype CBS H-25767; cultures ex-type CBS 154006 = FMR 20793).

Other specimens: SPAIN, Extremadura community, Badajoz province, Zújar River (38°24′31.4″ N 5°34′47.9″ W) (Granja de Torrehermosa), isolated from submerged plant debris, 12/11/2022, collected by Juan R. García Martínez, isolated by María Barnés Guirado (living culture FMR 20786).

Notes: *Neoanungitea torrehermosensis* differs from its closest species, *Neoanungitea eucalypti*, in producing smooth-walled (roughened in *N. eucalypti*) and much larger (60–250 × 4–8 µm in *N. torrehermosensis* vs. 30–160 × 4–6 µm in *N. eucalypti*) conidiophores. *Neoanungitea torrehermosensis* produces ramoconidia, which has not been reported for *N. eucalypti*. Moreover, the conidia of *N. torrehermosensis* are bigger than those of *N. eucalypti* (8.5–31 × 3–13 µm in *N. torrehermosensis* vs. 13–22 × 3.5–5 µm in *N. eucalypti*) and very variable in size (regularly cylindric-fusiform to navicular in *N. eucalypti*).

## 4. Discussion

Our survey of submerged, decaying plant debris in the Zújar River yielded a cultured assemblage of 49 isolates representing 24 taxa (Table 2), among which five species are newly described based on phylogenetic and phenotypic evidence. This combination of novelty and uneven taxon frequencies is reflected in a community structure dominated by a small number of recurrent saprobes (*Paraphaeosphaeria sporulosa* and *Hongkongmyces brunneosporus*) and a long tail of low-frequency taxa. This pattern is typical of freshwater-associated fungal communities retrieved by culturing, where a few competitively successful decomposers are repeatedly isolated while many taxa are detected sporadically. Together, these findings underscore how incomplete the inventory of freshwater fungi remains in Mediterranean river systems.

The genus *Polyscytalum* was established by Riess in 1853 to accommodate *Polyscytalum fecundissimum* [47]. According to Index Fungorum, to date, the genus includes 23 species [https://www.indexfungorum.org, accessed on 28 November 2025]; however, only eight of them have available molecular data. This genus has an extensive geographical distribution (e.g., the Americas, Australasia, Europe, and Malaysia). Regarding the species reported from Spain, *Polyscytalum pini-canariensis* and *Polyscytalum gracilisporum* have been isolated from the Canary Islands. Consequently, *Polyscytalum submersum* is the first report of the genus for the Iberian Peninsula [47,48,49,50,51,52,53]. The species of *Polyscytalum* are typically found on dead plant material/hosts belonging to the genera *Cedrus*, *Eucalyptus*, *Fagus*, *Grevillea*, *Nothofagus*, *Pinus*, *Quercus*, *Syzygium* and *Vaccinium* [47,48,49,50,51,52,53], and although *Polyscytalum submersum* was isolated from unidentified submerged plant debris, the Zújar River nearby harbors some of the plant genera reported as substrates for *Polyscytalum* spp., like *Eucalyptus*, *Quercus* and *Pinus* [13,14]. Despite being mostly isolated from dead plant material, *Polyscytalum* spp. are rarely reported as pathogenic [50,51,54,55]. Morphologically, the genus *Polyscytalum* can be divided into two main groups: one including those species producing acropetal chains of blastic cylindric conidia, and a second forming arthroconidia arising at the top of the verticillate conidiophores [51,56,57]. This morphological variability has led to the misplacement of some *Polyscytalum* species in genera like *Anungitea*, *Cylindrium*, and *Sympodiella*, and vice versa [47,50,52,53,57,58,59]. *Polyscytalum submersum*, belongs to the former group, sharing characteristics with the other species, such as the production of septate, hyaline to pale brown, cylindrical conidia, truncated at both ends. However, *P. submersum* is easily distinguishable from the rest of the species of the genus because of the production of anastomosing conidia throughout connectives [50,51,57].

Otherwise, the order *Magnaporthales* was established by Thongkantha et al. in 2009 to accommodate the family *Magnaporthaceae* [60]. Approximately 50% of all taxa in the family are pathogenic for monocotyledons, including *Pyricularia oryzae*, the rice blast fungus, *Nakataea oryzae*, the stem rot pathogen of rice, and *Gaeumannomyces graminis*, the most important rot pathogen of wheat and other cereals like barley, rye and triticale [61,62]. In 2014, Klaubauf et al. introduced two new families in the *Magnaporthales*, *Ophioceraceae* and *Pyriculariaceae*, to accommodate the genera *Ophioceras* and *Pyricularia*, respectively [32,63]. Currently, the genus includes 48 species, according to the Index Fungorum [https://www.indexfungorum.org, accessed on 28 November 2025], being typified by *O. dolichostomum*. This genus is characterized by producing immersed sub-carbonaceous perithecia, with globose bodies and long conic-cylindrical necks, unitunicate asci and septate filiform ascospores, being features in family delimitation [63,64,65,66]. Species of the genus have been isolated from Africa, Asia, Australasia, Central Africa, the Americas, the Netherlands and the UK [44,60,64,67,68,69,70,71,72,73,74,75,76]. *Ophioceras diversisporum* was isolated from a decomposing unidentified twig submerged in freshwater, a fact frequently reported for species of the genus; however, it represents the first report of the genus for Spain [44]. Although species of *Ophioceras* are generally characterized by the morphological features of their sexual morphs, *O. diversisporum* and *O. graminis* are the only species for which the sexual stage remains unknown. Additionally, only two species, *O. rhizomorpha* and *O. graminis*, are known to produce an asexual morph. *Ophioceras rhizomorpha* forms a didymobotryum-like anamorph, which differs markedly from the anamorph of *O. diversisporum*. Key differences include the presence of synnemata in *O. rhizomorpha* (absent in *O. diversisporum*). Furthermore, the conidiophores, conidiogenous cells, and conidia in O. *rhizomorpha* are dark brown, whereas those in *O. diversisporum* are hyaline. The conidia of *O. rhizomorpha* are septate, in contrast to the aseptate conidia of *O. diversisporum*. Notably, O. *diversisporum* also produces a synanamorph and appressoria, a fact not reported for *O. rhizomorpha* [44,77,78]. On the other hand, *O. graminis* produces lunate, allantoid to fusiform conidia and curved conidiogenous cells, along with hyphopodia, but lacks a synanamorph. In contrast, *O. diversisporum* produces ellipsoidal to slightly reniform, 2–3-guttulate conidia, and straight conidiogenous cells, appressoria, and a distinct synanamorph [78]. The species of *Ophioceras* are not known for pathogenic activity; in fact, some strains have been reported to exhibit antifungal properties against plant pathogens [79]. Conversely, although appressoria have been described in epiphytes, endophytes, and saprobes, they are primarily known as infective structures used by pathogens to penetrate host tissues [80]. Consequently, although there is currently no information regarding the pathogenicity of *O. diversisporum*, it may be considered a potential plant pathogen. Further studies are needed to clarify the nature of its relationship with host plants. Additionally, the phylogenetic analysis showed that *O. sichuanense* was placed in a basal, fully supported branch phylogenetically distant from the clade containing the remaining species of *Ophioceras*. This, along with its production of polyloculate pseudostromata with multiple necks, distinct from the uniloculate perithecial ascomata with a single long neck in other *Ophioceras* spp., led us to reclassify *O. sichuanense* into a new genus, *Protophioceras*.

The family *Conioscyphaceae* and the order *Conioscyphales* were stablished by Réblová et al. [81] to accommodate the genus *Conioscypha* [81] previously erected by Höhnel in 1904, with *Conioscypha lignicola* as a type species [82]. Nowadays, the genus contains 33 species, according to the Index Fungorum [https://www.indexfungorum.org, accessed on 28 November 2025]. Species of this genus have been reported from countries on every continent (including Spain) and are typically recovered from wood and other sorts of decaying plant material submerged in freshwater, conditions under which our novel species, *Conioscypha clavatispora*, was also found [64,83,84,85,86,87,88,89,90]. The species of this genus mostly present an asexual stage characterized by the production of cup-shaped, percurrently proloferating, multi-collaretted phialides from which the dematiaceous aseptate conidia are released [80,86,89]. *Conioscypha clavatispora* fits well within the genus based on both phenotypic and phylogenetic evidence. However, *C. clavatispora* is readily distinguishable from its closest relatives due to its phylogenetic divergence and distinctive conidial morphology. The conidia are ellipsoidal to clavate and large (9.5–22 × 4.5–10 μm), being ovoid, flamiform, navicular, or subellipsoidal and smaller (8.4–15 × 5.6–8.5 μm) in *C. varia*, the phylogenetical nearest species. Additionally, the conidia of *C. clavatispora* have a median pore and guttules, features not observed in *C. varia* [45,91,92]. Furthermore, due to the significant phylogenetic distance between *C. nakagirii*, *C. narathiwatensis* and the clade containing the rest of the species of *Conioscypha*, as well as its production of large, smooth-walled, turbinate to pyriform conidia, features not observed in any other species within the genus, we transferred *C. nakagirii* and *C narathiwatensis* to a new genus, *Protoconioscypha* [45,46,92].

The genus *Arachnopeziza*, erected by Fuckel in 1870 [93], currently includes 38 species, based on the Index Fungorum [https://www.indexfungorum.org, accessed on 28 November 2025], and it is included in the family *Arachnopezizaceae* together with the genera *Austropezia*, *Eriopezia* and *Parachnopeziza* [94]. There are two species of this genus traditionally considered the type species: *Arachnopeziza aurata* and *Arachnopeziza aurelia*. Both were described by Fuckel in 1870, *A. aurata* being the most widely accepted as type species [93,95]. The species of *Arachnopeziza* have a global distribution, being reported in Asia, Australasia, Europe and USA, and isolated from plants (both fresh and decaying) belonging to the genera *Arctostaphylos*, *Fagus*, *Juncus*, *Populus*, *Salix*, *Sphagnum*, *Calamagrostis*, *Festuca*, *Koeleria*, *Pinus*, and *Tilia*, as well on *Gramineae* [95,96,97,98,99,100,101]. *Arachnopeziza torrehermosensis*, our new species, represents the first report of the genus for Spain [95,96,97,98,99,100,101]. *Arachnopeziza torrehermosensis* was isolated from a freshwater submerged undetermined decomposing leaf, but the riparian flora of the Zújar River includes some plant genera from which species of the genus *Arachnopeziza* have been reported, such as *Festuca*, *Pinus*, *Populus*, and *Salix* [13]. Morphologically, the genus *Arachnopeziza* features uncolored to orange apothecia settled on a subiculum, with straight hairs and a hyaline excipulum, 8-spored cylindrical to claviform asci with an apical pore stained in blue with iodine solutions, and 1-7-septate ascospores [100]. Unfortunately, *A. torrehermosensis* did not produce fertile structures in natural subtract nor onto culture media at the lab; thus, it is not possible to perform a phenotypic comparison with other species of the genus. However, our phylogenetic analysis shows that *A. torrehermosensis* is placed in a well-supported branch within the genus and that the phylogenetic distance is sufficient to support its recognition as a distinct species within the genus, but not so long to suspect that it may belong to any other genus.

Lastly, the family *Microthyriaceae* was established by Saccardo in 1883 to accommodate the genus *Microthyrium* [66]. This family comprises 16 genera, eight of which reproduce asexually. The asexual stage is characterized by the production of micronematous to macronematous, mononematous, branched or unbranched conidiophores; the conidiogenous cells are terminal or integrated, mono- to polyblastic, and determinate or sympodial; the conidia are typically subcylindrical to ellipsoid or obclavate, verrucose, aseptate to multiseptate, and solitarily or in branched chains; the ramoconidia, when present, are aseptate, verrucose, and subcylindrical to fusoid-ellipsoid [102,103]. One of these asexual genera is the genus *Neoanungitea*, which was erected by Crous in 2017 [104]. Nowadays, the genus contains two species, *Neoanungitea eucalypti* and *Neoanungitea eucalyptorum* [https://www.indexfungorum.org, accessed on 28 November 2025], both isolated from Australia and from different species of *Eucalyptus* [104,105]. Consequently, our strains FMR 20793 and FMR 20786, in addition to representing a new species, also constitute the first report of the genus for Europe [104,105]. Although, as was mentioned above, the identity of the decomposing leaf from which both strains have been recovered remains unknown, we previously mentioned the presence of *Eucalyptus* near the Zújar River, a fact consistent with the substrate from which the other species of the genus have been isolated. Morphologically, the genus *Neoanungitea* is characterized by the production of erect, solitary, flexuous, subcylindrical conidiophores arising from brown stroma or from superficial hyphae. These conidiophores are multiseptate, thick-walled, roughened, and brown. The conidiogenous cells are terminal, subcylindrical, flat-tipped, thin-walled, finely roughened, and brown, forming a terminal rachis with several sympodial loci. The conidia are short, fusoid-ellipsoid, roughened, septate, pale brown, and arranged in branched chains, with obtuse ends and slightly thickened hila [104]. *Neoanungitea torrehermosensis* shares several diagnostic features with other species of the genus, yet it is distinguishable from its phylogenetically closest relative, *N. eucalypti*, by a suite of morphological differences. *Neoanungitea torrehermosensis* produces macronematous conidiophores straight at the base and slightly flexuous at the upper part, smooth-walled, and relatively large, measuring 60–250 × 4–8 µm. Its conidiogenous cells are cylindrical, barrel-shaped to ellipsoid, terminal or subterminal due to percurrent proliferation, occasionally geniculate at the apex, and comparatively short, ranging 21–37 × 3–6 µm. The conidia are cylindric, fusiform, or navicular, (1–)3-septate horizontally, occasionally with an oblique septum, truncated at the base or rarely at both ends, sometimes constricted at the septa, and relatively large, measuring 8.5–31 × 3–13 µm. In contrast, *N. eucalypti* (the phylogenetically nearest species) displays roughened and shorter conidiophores (30–160 × 4–6 µm), and terminal, subcylindrical, non-geniculate conidiogenous cells that are larger (20–60 × 4–7 µm). Its conidia are (0–)3-septate with no oblique septa, fusoid-ellipsoid, obtuse at both ends, not constricted at the septa, and shorter (13–22 × 3.5–5 µm). Additionally, *N. torrehermosensis* produces ramoconidia, which has not been observed in N. eucalypti. These distinct morphological characteristics, in combination with phylogenetic evidence, clearly support the recognition of *N. torrehermosensis* as a novel species within the genus [103,106].

## 5. Conclusions

This study provides the first culture-based survey of filamentous fungi associated with submerged, decaying plant debris in the Zújar River. From this substrate, we obtained 49 isolates representing 24 taxa, with the assemblage dominated by *Paraphaeosphaeria sporulosa* and *Hongkongmyces brunneosporus*. Importantly, seven isolates formed five well-supported, genetically distinct lineages that, together with diagnostic phenotypic characters, justify the description of five new species: *Arachnopeziza torrehermosensis*, *Conioscypha clavatispora*, *Neoanungitea torrehermosensis*, Ophioceras diversisporum, and *Polyscytalum submersum*. Beyond documenting local diversity, our results reinforce the value of a polyphasic approach for freshwater fungal systematics and provide taxonomic and phylogenetic evidence that supports a more robust classification of aquatic-associated ascomycete lineages.

## Figures and Tables

**Table 1:** Taxa and GenBank accession numbers of the molecular markers used in the phylogenetic analysis.

Taxon	Strain	GenBank Sequence Accession				
		ITS	LSU	*rpb*1	*rpb*2	*tef-*1α
*Adelosphaeria catenata*	CBS 138679^T^	NR_145396	NG_057081	-	KT278743	
*Amicodisca svrcekii*	TK7157	MT231647	MT231647	MT216583	-	MT434824
*Amicodisca virella*	SBRH828	MT231648	MT231648	MT216584	-	MT254577
*Anapleurothecium botulisporum*	CBS 132713^T^	KY853423	KY853483	-	-	-
*Anungitopsis lauri*	CBS 145067^T^	NR_161129	-	-	-	-
*Anungitopsis speciosa*	CBS 181.95	EU035401	EU035401	-	-	-
*Arachnopeziza araneosa*	PDD 59117	MH578555	-	-	-	-
*Arachnopeziza araneosa*	PDD 74085	MH578557	-	-	-	-
*Arachnopeziza aurelia*	KUS-F51520	JN033409	JN086712	-	-	-
*Arachnopeziza aurelia*	CBS 117.54	MH857261	MH868796	-	-	-
*Arachnopeziza aurata*	TUR 179456	MT231649	MT231649	-		MT241676
*Arachnopeziza delicatula*	TK7076	MT231650	MT231650	MT216585	--	MT254567
*Arachnopeziza delicatula*	JK14051801	MT231651	MT231651	MT216586	-	MT241687
*Arachnopeziza estonica*	SH15/38T	MT231657	MT231657	MT216589	-	MT241693
*Arachnopeziza estonica*	TL210	MT231658	MT231658	MT216590	-	MT241677
*Arachnopeziza japonica*	SH06/03	MT231661	MT231661	MT216593	-	MT241683
*Arachnopeziza japonica*	RI194	MT231662	MT231662	MT216594	-	MT241684
*Arachnopeziza leonina*	TK7101	MT231666	MT231666	MT216598	-	MT241694
*Arachnopeziza leonina*	KH.15.23	MT231665	MT231665	MT216597	-	MT254566
*Arachnopeziza obtusipila*	TNS-F12768	JN033445	JN086746	-	-	-
*Arachnopeziza obtusipila*	TNS-F12769	JN033446	JN086747	-	-	-
*Arachnopeziza ptilidiophila*	TK7287^T^	MT231668	MT231668	MT216600	-	-
*Arachnopeziza ptilidiophila*	TK7290	MT231670	MT231670	-	-	-
*Arachnopeziza ptilidiophila*	AL51	MT231671	MT435517	MT216603	-	MT241681
*Arachnopeziza* sp.	TM285	MT435517	MT435517	MT216603	-	MT241681
*Arachnopeziza sphagniseda*	RI267	MT231676	MT231676	MT216608	-	-
*Arachnopeziza sphagniseda*	TUR 178046	MT231677	MT231677	-	-	-
***Arachnopeziza torrehermosensis***	**FMR 20792^T^**	**PV029860**	**PV029867**	**PV014926**	-	**PV014925**
*Arachnopeziza trabinelloides*	GJO0071771	MT231679	MT231679	MT216609	-	MT241697
*Ascotaiwania lignicola*	NIL00005	HQ446341	HQ446364	-	HQ446419	-
*Bactrodesmiastrum pyriform*	FMR 10747^T^	NR_152536	FR870265	-	-	-
*Bactrodesmium pallidum*	FMR 11345	KY853425	KY853485	-	-	-
*Bambusicularia brunnea*	CBS 133599^T^	NR_145387	-	-	-	-
*Barretomyces calatheae*	CBS 129274	MH865202	-	KM485045	-	-
*Canalisporium caribense*	SS03683	GQ390284	GQ390268	-	HQ446421	-
*Canalisporium elegans*	SS00895	GQ390286	GQ390271	-	-	-
*Canalisporium pulchrum*	SS03982	GQ390292	GQ390277	-		-
*Ceratosphaeria aquatica*	MFLU 18-2323^T^	NR_168793	NG_068628	-	-	-
*Ceratosphaeria flava*	MFLUCC 15-0058^T^	OP377883	OP377969	-	-	-
*Ceratosphaeria lampadophora*	CBS 125415	MH863598	MH875074	-	-	-
*Ceratosphaeria lignicola*	MFLU 18-1457^T^	NR_168794	MK835813	-	-	-
*Ceratosphaeria phialidica*	SMH1643^T^		AY346295	-	-	-
*Ceratosphaeria suthepensis*	PDD 76762		NG_079624	-	-	-
*Ceratosphaeria yunnanensis*	KUMCC 21-0013^T^	NR_184381	NG_149017	-	-	-
*Conioscypha aquatica*	MFLUCC 18-1333^T^	MK878383	MK835857	-	MN194030	-
*Conioscypha bambusicola*	JCM 7245^T^	NR_154660	NG_059037	-	-	-
*Conioscypha boutwelliae*	CBS 144928^T^	LR025182	LR025183	-	-	-
*Conioscypha breviconiophora*	KUNCC 24-17971^T^	PQ168253	PQ152641	-	-	-
*Conioscypha breviconiophora*	KUNCC 24-18128	PQ168252	PQ152640	-	-	-
*Conioscypha chiangmaiense*	MFLUCC 21-0158^T^	NR_182481	NG_149018	-	-	-
***Conioscypha clavatispora***	**FMR 20788^T^**	**PV029859**	**PV029866**	-	**PV014924**	-
***Conioscypha clavatispora***	**FMR 20897**	**PV029858**	**PV029865**	-	**PV014923**	-
*Conioscypha hoehnelii*	FMR 11592	KY853437	KY853497	-	-	-
*Conioscypha japonica*	CBS 387.84^T^	-	AY484514	-	JQ429259	-
*Conioscypha lignicola*	CBS 335.93	-	AY484513	-	JQ429260	-
*Conioscypha minutiellipsoidea*	CBS 112523^T^	NR_175115	NG_078663	-	-	-
*Conioscypha minutiellipsoidea*	MFLU 17-1724	MN513033	MN512342	-	MT150077	-
*Conioscypha minutispora*	FMR 11245^T^	NR_137847	KF924559	-	-	-
*Conioscypha motuoensis*	KUNCC 10471^T^	OR458372	OR473154	-	-	-
*Conioscypha motuoensis*	KUNCC 10485	PP087960	PP087963	-	-	-
*Conioscypha muchuanensis*	CGMCC 3.27448^T^	PQ067931	PQ067761	-	PQ186983	-
*Conioscypha obovoidea*	MFLU 24-0284^T^	PQ570854	PQ570871	-	-	-
*Conioscypha peruviana*	CBS 137657	-	NG_058867	-	-	-
*Conioscypha pleiomorpha*	CBS 138110^T^	KY853438	KY853498	-	-	-
*Conioscypha punctiformis*	HKAS 124553^T^	PP657272	PP657307	-	PP887801	-
*Conioscypha sichuanensis*	CGMCC 3.24356^T^	NR_197543	NG_243946	-	OR862128	-
*Conioscypha subglobosa*	KUNCC 10478^T^	OR458379	PQ152638	-	-	-
*Conioscypha subglobosa*	KUNCC 24-18082	-	PQ152639	-	-	-
*Conioscypha submersa*	MFLU 18-1639^T^	MK878382	MK835856	-	-	-
*Conioscypha synnemata*	HKAS 136889^T^	PQ570853	PQ570870	-	-	-
*Conioscypha tenebrosa*	MFLU 19-0688^T^	MK804506	MK804508	-	MK828514	-
*Conioscypha tenebrosa*	MFLU 19-0687	MK804507	MK804509	-	MK828515	-
*Conioscypha varia*	CBS 436.70^T^	MH859785	MH871548	-	-	-
*Conioscypha varia*	CBS 602.70	MH859868	MH871654	-	-	-
*Conioscypha verrucosa*	MFLUCC 18-0419^T^	MN061350	NG_068893	-	MN061668	-
*Conioscypha xizangensis*	HKAS 130588^T^	OR674790	OR674849	-	OR684565	-
*Conioscypha yunnanensis*	KUNCC23-13319^T^	OR234669	OR478379	-	OR487158	-
*Conioscypha yunnanensis*	KUNCC23-13172	OR478183	OR478380	-	OR487157	-
*Eriopezia caesia*	TK7005	MT231685	MT231685	MT216615	-	MT241673
*Gaeumannomyces graminis*	CPC 26020	KX306498	KX306568	KX306633	-	-
*Helicoascotaiwania farinosa*	DAOM 241947	JQ429145	JQ429230	-	-	-
*Isthmomyces oxysporus*	YMF1.04513^T^	MF740793	MF740793	-	-	-
*Keqinzhangia aquatica*	YMF1.04262^T^	MK569507	MK569507	-	-	-
*Macgarvieomyces borealis*	CBS 461.65^T^	NR_145384	NG_058088	KM485070	-	-
*Magnaporthiopsis cynodontis*	CBS 141700^T^	NR_172813	NG_075193	-	-	-
*Melanotrigonum ovale*	CBS 138743^T^	NR_145397	NG_058197	-	KT278745	-
*Microthyrium buxicola*	MFLUCC 15-0212^T^	-	KT306551	-	-	-
*Microthyrium buxicola*	MFLUCC 15-0213	-	KT306552	-	-	-
*Microthyrium chinens*	HKAS 92487^T^	-	NG_241900	-	-	-
*Microthyrium fici-septicae*	MFLU 19-2789^T^	-	NG_079545	-	-	-
*Microthyrium ilicinum*	CBS 143808	-	MG844151	-	-	-
*Microthyrium macrosporum*	CBS 143810	-	MG844159	-	-	-
*Microthyrium microscopicum*	CBS 115976	OL739259	OL739259	-	-	-
*Microthyrium propagulensis*	IFRDCC 9037^T^	-	NG_060339	-	-	-
*Nakataea oryzae*	CBS 332.53	MH857230	MH868767	KM485083	-	-
*Neoanungitea eucalypti*	CBS 143173^T^	MG386031	MG386031	-	-	-
*Neoanungitea eucalyptorum*	CBS 146028^T^	NR_166310	NR_166310	-	-	-
***Neoanungitea torrehermosensis***	**FMR 20793^T^**	**PV029861**	**PV029868**	**-**	**-**	**-**
***Neoanungitea torrehermosensis***	**FMR 20786**	**PV029862**	**PV029869**	**-**	**-**	**-**
*Neoascotaiwania limnetica*	CBS 126576	KY853452	KY853513	-	-	-
*Neoascotaiwania terrestris*	CBS 142291^T^	NR_154260	NG_058460	-	-	-
*Neocordana musicola*	CPC 11225^T^	NR_154266	-	-	-	-
*Neopyricularia commelinicola*	CBS 128308^T^	NR_154226	NG_058112	KM485087	-	-
*Nothoanungitopsis urophyllae*	CPC 38059^T^	MW883433	MW883433	-	-	-
*Omnidemptus affinis*	ATCC 200212^T^	NR_154292	NG_059478	JX134728	-	-
*Ophioceras aquaticum*	IFRDCC 3091^T^	NR_165842	NG_067778	-	-	-
*Ophioceras aquaticum*	MFLUCC 16-0906	MK828611	MK835810	-	-	-
*Ophioceras aseptatum*	KUNCC:23-14570^T^	OR589313	OR600961	-	-	-
*Ophioceras castillensis*	SMH1865	-	EU527997	-	-	-
*Ophioceras chiangdaoense*	CMU 26633	-	NG_066356	-	-	-
*Ophioceras chiangdaoense*	MFLU 19-2730	-	MW114438	-	-	-
*Ophioceras commune*	HKAS 92587	MH795814	MH795819	-	-	-
*Ophioceras commune*	M91	JX134675	JX134687	JX134729	-	-
*Ophioceras cylindrosporum*	KUNCC:23-13706^T^	OR589314	OR600962	-	-	-
***Ophioceras diversisporum***	**FMR 20787^T^**	**PV029857**	**PV029864**	**PV014922**	**-**	**-**
*Ophioceras dolichostomum*	CBS 114926	JX134677	JX134689	JX134731	-	-
*Ophioceras dolichostomum*	HKUCC3936	-	DQ341508	-	-	-
*Ophioceras ficinum*	MFLU 19-2751^T^	-	NG_079552	-	-	-
*Ophioceras ficinum*	NCYU 19-0022	-	MW114437	-	-	-
*Ophioceras freycinetiae*	CBS 146781^T^	NR_173031	NG_076724	-	-	-
*Ophioceras graminis*	CGMCC3.20904^T^	MW479093	-	MW482855	-	-
*Ophioceras graminis*	YNE00717	MW479094	-	MW482856	-	-
*Ophioceras guizhouensis*	MFLU 18-2277^T^	NR_191278	-	-	-	-
*Ophioceras hongkongense*	HKUCC3624^T^	-	NG_088007	-	-	-
*Ophioceras junci*	CBS 148450^T^	NR_175243	OK663789	OK651155	-	-
*Ophioceras junci*	CPC 42235	OK664751	OK663790	OK651156	-	-
*Ophioceras leptosporum*	CBS 894.70^T^	NR_111768	NG_057959	JX134732	-	-
*Ophioceras leptosporum*	CPC 39147	MW883435	MW883827	-	-	-
*Ophioceras rhizomorpha*	GKM 1262^T^	-	NG_153826/EU527998	-	-	-
*Ophioceras submersum*	MFLUCC 18-0211^T^	-	NG_068627	-	-	-
*Ophioceras thailandense*	MFLUCC 15-0603^T^	OP377882	NG_243762	-	-	-
*Ophiostoma ainoae*	CBS 205.83^T^	NR_147579	NG_067421	-	-	-
*Ophiostoma piliferum*	CBS 158.74	-	DQ470955	DQ471147	-	-
*Paramirandina aquatica*	GZCC 19-0408^T^	OQ025199	OQ025199	-	-	-
*Paramirandina cymbiformis*	HKAS 112619^T^	-	NG_243192	-	-	-
*Phaeoisaria clematidis*	CBS 149173	ON811520	ON811578	-	-	-
*Phaeoisaria fasciculata*	CBS 127885^T^	NR_145395	NG_064241	-	-	-
*Phragmocephala stemphylioides*	DAOM 673211	KT278730	KT278717	-	-	-
*Plagiascoma frondosum*	CBS 139031^T^	-	NG_058198	-	KT278749	-
*Pleurotheciella centenaria*	DAOM 229631^T^	NR_111709	NG_060098	-	JQ429265	-
*Pleurotheciella rivularia*	CBS 125238^T^	NR_111711	NG_057950	-	JQ429263	-
*Pleurothecium recurvatum*	CBS 138747	-	KT278714	-	-	-
*Pleurothecium recurvatum*	CBS 138686	KT278727	KT278715	-	-	-
*Pleurothecium semifecundu*	CBS 131271^T^	NR_111710	NG_057951	-	JQ429270	-
*Polyscytalum chilense*	CBS 143387^T^	NR_158958	MH107954	-	-	-
*Polyscytalum eucalyptorum*	CBS 137967	NR_132904	KJ869176	-	-	-
*Polyscytalum fecundissimum*	CBS 100506	EU035441	EU035441	-	-	-
*Polyscytalum grevilleae*	CBS 141282^T^	NR_154719	KX228304	-	-	-
*Polyscytalum neofecundissimum*	CBS 143390^T^	NR_158959	NG_066207	-	-	-
*Polyscytalum pini-canariensis*	CBS 146819^T^	NR_171768	NG_074496	-	-	-
*Polyscytalum pinicola*	CPC 36759^T^	NR_170062	NG_074425	-	-	-
*Polyscytalum vaccinii*	CPC 39935	OK664709	OK663748	-	-	-
***Polyscytalum submersum***	**FMR 20795^T^**	**PV029856**	**PV029863**	**-**	**-**	**-**
*Protoconioscypha nakagirii*	BCC77658^T^	KY859266	KU509985	-	KU513952	-
*Protoconioscypha nakagirii*	BCC77659	KY859267	OR478379	-	KU513953	-
*Protoconioscypha narathiwatensis*	MFLUCC 24-0581^T^	PV271887	PV271926	-	PV340529	-
*Protoconioscypha narathiwatensis*	MFLUCC 24-0582	PV271888	PV271927	-	PV340534	-
*Protophioceras sichuanense*	KUMCC 20-0213^T^	MT995045	MT995046	-	-	-
*Protophioceras sichuanense*	HKAS 107677	MW057782	MW057779	-	-	-
*Proxipyricularia zingiberis*	CBS 303.39	KM484871	KM484989	KM485092	-	-
*Pseudocorniculariella guizhouensis*	GZCC 19-0513^T^	OQ025200	OQ025200	-	-	-
*Pseudohalonectria aurantiaca*	MFLUCC 15-0379	OP377881	-	-	-	-
*Pseudohalonectria lignicola*	M95	JX134679	JX134691	JX134733	-	-
*Pseudohalonectria lutea*	CBS 126574	MH864160	-	-	-	-
*Pyricularia grisea*	CBS 138707^T^	NR_172230	MH877665	-	-	-
*Pyriculariomyces asari*	CPC 27444^T^	NR_145407	NG_058246	KX228368	-	-
*Savoryella bambusicola*	CGMCC 3.23775	OQ428269	OQ428261	-	OQ437185	-
*Spirosphaera beverwijkiana*	CBS 469.66^T^	HQ696657	HQ696657	-	-	-
*Spirosphaera beverwijkiana*	CBS 470.66	MH858860	MH870500	-	-	-
*Spirosphaera beverwijkiana*	CBS 474.66	MH858861	MH858861	-	-	-
*Spirosphaera carici-graminis*	CBS 617.97^T^	NR_171738	-	-	-	-
*Spirosphaera floriformis*	CBS 402.52^T^	NR_138376	MH868632	-	-	-
*Spirosphaera floriformis*	CBS 403.52	MH857098	MH868633	-	-	-
*Spirosphaera minuta*	CBS 475.66^T^	-	NG_064056	-	-	-
*Spirosphaera minuta*	CBS 476.66	HQ696659	MH870503	-	-	-
*Spirosphaera minuta*	CBS 477.66	MH858862	MH870504	-	-	-
*Spirosphaera minuta*	CBS 498.66	MH858870	MH870511	-	-	-
*Sterigmatobotrys macrocarpa*	CBS 113468	JQ429154	-	-	JQ429271	-
*Subulispora biappendiculata*	CBS 121489	MH863112	MH874667	-	-	-
*Subulispora britannica*	ICMP 14767	EF029198	-	-	-	-
*Subulispora procurvata*	CBS 567.71	MH860265	-	-	-	-
*Subulispora rectilineata*	CBS 568.71	MH860266	MH872029	-	-	-
*Sympodiella multiseptata*	CBS 566.71	MH860264	MH860264	-	-	-
*Triscelophorus anisopteroideus*	YMF1 04267^T^	MK569511	MK569511	-	-	-
*Utrechtiana roumeguerei*	CBS 128780	MH865092	-	KM485047	-	-
*Xenopyricularia zizaniicola*	CBS 133593^T^	NR_185354	-	KM485161	-	-
*Zeloasperisporium ficusicola*	MFLUCC 15-0221^T^	-	NG_059598	-	-	-
*Zeloasperisporium hyphopodioides*	CBS 218.95^T^	EU035442	EU035442	-	-	-

^T^ = Ex-type strains. In bold, sequences generated in this study. BCC = BIOTEC Culture Collection (Thailand). CBS = Centraalbureau voor Schimmelcultures, Fungal Biodiversity Centre (the Netherlands). CGMCC = China General Microbiological Culture Collection Center (China). CPC = Collection of P.W. Crous (CBS; the Netherlands). DAOM = National Mycological Herbarium, Department of Agriculture, Ottawa (Canada). FMR = Faculty of Medicina, Reus culture collection (Spain). GKM = G.K. Mugambi personal culture collection (China and Thailand). GJO = Universalmuseum Joanneum (Austria). GZAAS = Guizhou Academy of Agriculture Sciences (China). HKAS = Herbarium of Cryptogams, Kunming Institute of Botany Academia Sinica (China). GZCC = Guizhou Culture Collection (China). HKUCC = University of Hong Kong Culture Collection (China). ICMP = International Collection of Microorganisms (New Zealand). IFRDCC = International Fungal Research & Development Centre Culture Collection (China). JCM = Japan Collection of Microorganisms (Japan). JK and SBRH = strains in the University of Turku (Finland) and in the Swedish Museum of Natural History (Sweden). KAS = Kunming Institute of Botany Academia Sinica culture collection (China). KUMCC = Kumamoto University Microbial Culture Collection (Japan). KUNCC = Culture Collection Center, Kunming (China). MFLUCC = Mae Fah Luang culture collection (Thailand). NIL = strain sin BCC. TK = Tomsk State University (Russia). YMF = Key Laboratory of Industrial Microbiology and Fermentation Technology of Yunnan (China). YNE = strains in CGMCC.

**Table 2:** Fungal strains with less than 98% identity (ITS) compared to the nucleotide sequence of the molecular marker of the closest species.

Strain	Locus	Closest Species and Strain	% Identity *	Identical/Total **	Gaps	GenBank Accession	Order
FMR 20795	ITS	*Polyscytalum eucalyptorum* CBS 137967	95.68	339/353	1	JF449466	*Xylariales*
		*Polyscytalum chilense* CBS 143387	94.70	482/509	3	NR_158958	
	LSU	*Polyscytalum pinicola* CPC 36759 *Polyscytalum chilense* CBS 143387	99.38	798/803	1	NG_074425	
			99.29	834/840	2	MH107954	
FMR 20787	ITS	*Ophioceras thailandense* MFLUCC 15-0603	88.69	345/389	13	NR_197509	*Magnaportales*
	LSU	*Ophioceras aquaticum* IFRDCC 3091	97.00	777/801	0	NG_067778	
	*rpb*1	*Ophioceras dolichostomum* CBS 114926	83.50	522/625	7	JX134731	
FMR 20788	ITS	*Conioscypha submersa* MFLU 18-1639	93.18	205/220	2	NR_168820	*Conioscyphales*
	LSU	*Conioscypha varia* CBS 436.70	97.93	805/822	4	MH871548	
	*rpb*2	*Conioscypha verrucosa* MFLU 18-1503	88.36	774/876	4	MN061668	
FMR 20897	ITS	*Conioscypha submersa* MFLU 18-1639	93.18	205/220	2	NR_168820	*Conioscyphales*
	LSU	*Conioscypha varia* CBS 436.70	97.89	788/805	4	MH871548	
	*rpb*2	*Conioscypha verrucosa* MFLU 18-1503	88.55	781/882	4	MN061668	
FMR 20792	ITS	*Arachnopeziza delicatula* JK14051801	97.12	512/525	4	HM030576	*Helotiales*
	LSU	*Arachnopeziza delicatula* TK7152	98.84	682/690	1	MT231656	
	*tef-*1α	*Arachnopeziza leonina* KH.15.23	94.50	790/836	0	MT254566	
	*rpb*1	*Arachnopeziza delicatula* JP6655	93.07	658/707	2	MT216587.1	
FMR 20786	ITS	*Neoanungitea eucalypti* CBS 143173	94.40	354/375	9	NR_156383.1	*Xylariales* *
	LSU	*Neoanungitea eucalypti* CBS 143173	99.76	831/833	1	MG386031.2	
FMR 20793	ITS	*Neoanungitea eucalypti* CBS 143173	94.37	620/657	14	NR_156383.1	*Xylariales* *
	LSU	*Neoanungitea eucalypti* CBS 143173	99.76	830/832	0	MG386031.2	

FMR = Faculty of Medicine, Reus culture collection, Spain. * Based on BLAST search results. ** Number of identical nucleotides over the total sequence.

## Data Availability

DNA sequence data generated in this study have been deposited in GenBank under accession numbers (see Table 1). The alignments, tree files, and associated metadata have been deposited in Zenodo (DOI: 10.5281/zenodo.17955457). The ex-type and reference cultures are deposited in CBS and FMR (see Table 1 and Table 2).

## Abbreviations

The following abbreviations are used in this manuscript:

*act*	Actin
BCC	BIOTEC Culture Collection
BI	Bayesian Inference
BLAST	Basic Local Alignment Search Tool
BS	Bootstrap Support
CBS	Centraalbureau voor Schimmelcultures, Westerdijk Fungal Biodiversity Institute
CGMCC	China General Microbiological Culture Collection Center
comb. nov.	Novel Combination
CPC	Culture Collection of P.W. Crous
DAOM	National Mycological Herbarium, Department of Agriculture Ottawa
FMR	Faculty of Medicine of Reus
GJO	Universalmuseum Joanneum
GKM	G.K. Mugambi personal culture collection
GZAAS	Guizhou Academic of Agriculture Sciences
GZCC	Guizhou Culture Collection
HKAS	Herbarium of the Kunming Institute of Botany
HKUCC	University of Hong Kong Culture Collection
HVVV	Personal collection of Wayne Pitt from Vitis vinifera
ICMP	International Collection of Microorganisms
IFRDCC	International Fungal Research & Development Centre Culture Collection
ITS	Internal Transcribed Spacers
JCM	Japan Collection of Microorganisms
JK	University of Turku
KAS	Kunming Institute of Botany Academia Sinica Culture Collection
KUMCC/KUNCC	Kunming Institute of Botany Culture Collection
LSU	D1–D2 Domains of the 28S nrRNA
MCMC	Markov–Monte Carlo Chains
MEA	2% Malt Extract Agar
MEGA	Molecular Evolutionary Genetics Analysis
MFLU	Herbarium of the Mae Fah Luang University
MFLUCC	Culture Collection of the Mae Fah Luang University
mm	Millimeter
ML	Maximum Likelihood
MLI	Maximum Level of Identity
MUSCLE	Comparison of Multiple Sequences by Expectation
NIL	Strains in BCC
OA	Oatmeal Agar
PCA	Potato Carrot Agar
PCR	Polymerase Chain Reaction
PDA	Potato Dextrose Agar
PP	Posterior Probability
*rpb*1	RNA Polymerase II Subunit 1
*rpb*2	RNA Polymerase II Subunit 2
SBRH	Swedish Museum of Natural History
sp.	Species
spp.	Species (plural)
*tef-*1α	Translation Elongation Factor 1α
TK	Tomsk State University
YMF	Key Laboratory of Industrial Microbiology and Fermentation Technology of Yunnan
YNF	Strains in CGMCC

## Appendix A

**Table A1:** Main data of the fungal strains from submerged decaying plant material in the Zújar River (Extremadura, Spain).

Living Strains (and Holotype)	Identification	Molecular Markers	Sequence Identity (%) *	GenBank Accession Number
FMR 20899	*Acremonium sclerotigenum*	ITS	100%	MF075142
		LSU	100%	MH868961
**FMR 20792** **(CBS H-25766)**	***Arachnopeziza torrehermosensis***	**ITS**	**97.12%**	**MT231651**
		**LSU**	**98.84%**	**MT231655**
		***tef-*** **1α**	**94.50%**	**MT254566**
		***rpb*** **1**	**93.07%**	**MT216587**
FMR 20502	*Bartalinia robillardoides*	ITS	100%	NR_126145
		LSU	99.71%	EU552102
FMR 20796	*Bartalinia robillardoides*	ITS	100%	NR_126145
		LSU	100%	EU552102
FMR 20811	*Cladosporium ramotenellum*	*tef-*1α	98.44%	KT600533
		*act*	99.39%	KT600622
**FMR 20788 = CBS 154005 (CBS H-25765)**	***Conioscypha clavatispora***	**ITS**	**93.18%**	**NR_168821**
		**LSU**	**97.93%**	**MH871548**
		***rpb*** **2**	**88.36%**	**MN061668**
**FMR 20897**	***Conioscypha clavatispora***	**ITS**	**93.18%**	**NR_168821**
		**LSU**	**97.89%**	**MH871654**
		***rpb*** **2**	**88.55%**	**MN061668**
FMR 20789	*Dichotomopilus indicus*	*tub*2	99.40%	JF772451
FMR 20781	*Fusarium oxysporum*	ITS	99.80%	OM977105
		LSU	99.54%	MH876100
		*tef*-1α	98.74%	JQ429355
FMR 20552	*Hongkongmyces brunneosporus*	LSU	99.57%	MW004646
FMR 20798	*Hongkongmyces brunneosporus*	LSU	100%	MW004643
FMR 20799	*Hongkongmyces brunneosporus*	LSU	100%	MW004645
FMR 20803	*Hongkongmyces brunneosporus*	LSU	99.76%	MW004646
FMR 20805	*Hongkongmyces brunneosporus*	LSU	99.51%	MW004645
FMR 20810	*Hongkongmyces brunneosporus*	LSU	99.76%	MW004644
FMR 20898	*Hongkongmyces brunneosporus*	LSU	99.86%	MW004643
FMR 20553	*Hongkongmyces snookiorum*	ITS	99.49%	OR004657
		LSU	99.52%	MW757254
FMR 20780	*Hongkongmyces snookiorum*	ITS	99.35%	MH161189
FMR 20813	*Lecanicillium psalliotae*	ITS	99.44%	JN797793
FMR 20801	*Lecanicillium saksenae*	ITS	99.64%	PP620758
		LSU	100.00%	MH861374
**FMR 20786 = CBS 154006 (CBS H-25767)**	***Neoanungitea torrehermosensis***	**ITS**	**94.68%**	**NR_156383**
		**LSU**	**99.76%**	**MG386031**
**FMR 20793**	***Neoanungitea torrehermosensis***	**ITS**	**94.70%**	**NR_156383**
		**LSU**	**99.76%**	**MG386031**
**FMR 20787 = CBS 154004 (CBS H-25764)**	***Ophioceras diversisporum***	**ITS**	**88.69%**	**NR_197509**
		**LSU**	**97.00%**	**NG_067778**
		***rpb*** **1**	**83.52%**	**JX134731**
FMR 20541	*Paraphaeosphaeria sporulosa*	ITS	100%	JX496045
		LSU	100%	JX496175
FMR 20665	*Paraphaeosphaeria sporulosa*	ITS	100%	JX496114
FMR 20783	*Paraphaeosphaeria sporulosa*	ITS	100%	JX496110
FMR 20785	*Paraphaeosphaeria sporulosa*	ITS	100%	JX496108
		LSU	100%	JX496187
FMR 20791	*Paraphaeosphaeria sporulosa*	ITS	100%	MH854865
FMR 20800	*Paraphaeosphaeria sporulosa*	ITS	99.82%	JX496084
		LSU	99.79%	MH871696
FMR 20802	*Paraphaeosphaeria sporulosa*	ITS	100%	JX496045
FMR 20804	*Paraphaeosphaeria sporulosa*	ITS	99.82%	JX496114
		LSU	100%	MH866362
FMR 20809	*Paraphaeosphaeria sporulosa*	ITS	99.85%	JX496114
FMR 20895	*Paraphaeosphaeria sporulosa*	LSU	98.71%	JX496186
FMR 20504	*Paraphaeosphaeria sporulosa*	ITS	100%	JX496108
FMR 20500	*Parascedosporium putredinis*	ITS	100%	MN047111
FMR 20669	*Phialophora americana*	LSU	100%	MH877539
FMR 20794	*Phialophora americana*	*tef-*1α	98.12%	MH048681
FMR 20797	*Plectosphaerella cucumerina*	ITS	99.81%	MH862743
		LSU	100%	MH874350
**FMR 20795 = CBS 154003 (CBS H-25763)**	***Polyscytalum submersum***	**ITS**	**95.68%**	**KJ869118**
		**LSU**	**99.38%**	**NG_074425**
FMR 20812	*Prosthemium neobetulinum*	ITS	98.77%	MH856774
FMR 20807	*Stagonospora pseudoperfecta*	ITS	100%	MK442625
		LSU	100%	NG_059399
FMR 20782	*Sympoventuria capensis*	ITS	98.89%	NR_121323
FMR 20790	*Sympoventuria capensis*	ITS	99.10%	NR_121323
FMR 20660	*Talaromyces muroii*	*tub*2	99.74%	KJ865727
FMR 20676	*Talaromyces muroii*	*tub*2	99.73%	KM066151
FMR 20505	*Typhicola typharum*	ITS	99.85%	KF251192
FMR 20663	*Typhicola typharum*	ITS	99.27%	MK442590
FMR 20806	*Typhicola typharum*	ITS	99.57%	KF251192
		LSU	99.88%	MK442530
FMR 20501	*Vermiculariopsiella dichapetali*	ITS	100%	MH107924
		LSU	100%	MH107970

CBS-H = CBS Herbarium, Westerdijk Fungal Biodiversity Institute, Utrecht, the Netherlands. FMR = Faculty of Medicine, Reus culture collection, Spain. Data corresponding to a putatively new species are shown in bold. * Using BLAST+ version 2.17.0 (https://blast.ncbi.nlm.nih.gov/Blast.cgi, accessed on 28 November 2025) and Mycobank (https://www.mycobank.org/Pairwise_alignment, accessed on 28 November 2025).
